# Development of 1,5-Diaryl-Pyrazole-3-Formate Analogs as Antifungal Pesticides and Their Application in Controlling Peanut Stem Rot Disease

**DOI:** 10.3389/fmicb.2021.728173

**Published:** 2022-01-04

**Authors:** Yihui Ma, Jun Yang, Dangwei Yang, Guangyu Qin, Junhuai Zu

**Affiliations:** Plant Protection Institute, Henan Academy of Agricultural Sciences, Zhengzhou, China

**Keywords:** 1,5-diaryl-pyrazole-3-formate, antifungal pesticides, peanut stem rot disease, synthesis, *Sclerotium rolfsii*

## Abstract

Stem rot disease caused by *Sclerotium rolfsii* is one of the destructive diseases in peanut and poses a big risk to peanut production. Current fungicides in the market have not provided satisfactory control efficacy and thus called for novel fungicides with different structures as an alternative treatment strategy. Our previously developed phenylpyrazole compound **3c** demonstrated modest inhibitory effect against *S. rolfsii.* The following structure modification identified an unreported compound **6,** which bears a 3-chloropyridinyl moiety as the most prominent derivative with an IC_50_ of 12 μg/ml in potato dextrose agar (PDA) assay, higher than those of 0.8 and 1.8 μg/ml associated with thifluzamide and tebuconazole, respectively. However, compound **6** showed similar controlling effects to those of thifluzamide and tebuconazole in field study. This study underscores the potential of 1,5-diaryl-pyrazole-3-formate as an antifungal candidate for stem rot disease management.

## Graphical Abstract

3-Chloropyridinyl moiety grants compound **6** with comparable antifungal effect to tebuconazole and thifluzamide against *Sclerotium rolfsii* in a dose-dependent manner.

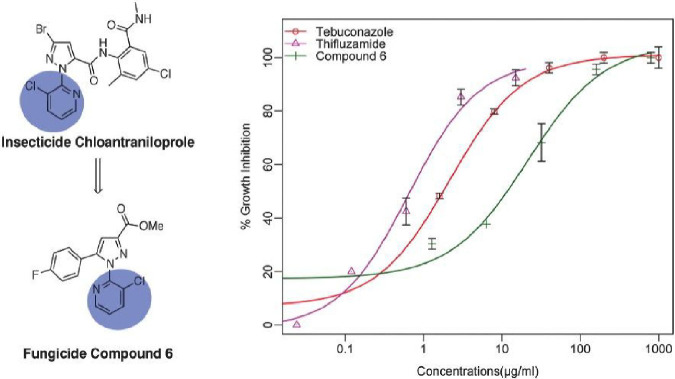

## Introduction

Stem rot disease is one of the destructive diseases in peanuts and leads to significant loss to peanut yields (Punja, 1985). The disease commonly occurs in most major peanut-growing areas of the world (Bowen, 2003; Thiessen and Woodward, 2012). Pathologically, stem rot disease is caused by *Sclerotium rolfsii* (= *Athelia rolfsii*), a pathogenic fungus whose growing mycelia has thick, white silk-like hyphae (also called “white silk-like disease” in China). The mycelia can spread out on the soil surface and reach for new hosts. In the wet season, the disease is more likely to develop into a severe outbreak (Culbreath, 1992; Davis et al., 1996) in sandy soils (Thiessen and Woodward, 2012). Once infected, the peanuts would present symptoms such as a yellowing and wilting of the top part of the plant at the early stage before drying up within a few days of symptom expression due to the lack of water supply in high temperatures. In recent years, peanut stem rot disease has been developing rapidly throughout the Henan province of central China for the growing planting area and continuous cropping, and is identified as a major challenge for peanut production (Tang, 2009). Cultural controlling strategies such as crop rotation are generally not well adopted by peanut growers for the economic concerns and fungicide treatment still represents an effective management strategy for disease control. However, stem rot disease has not been successfully managed with the current commercial fungicides (Grichar, 1995; Hagan et al., 2004). Thus, pursuit of novel fungicides with unique structure and a high level of activity for effective control of stem rot disease is much needed.

Pyrazole, a five-membered heterocyclic ring with two adjacent nitrogen atoms, is a frequently present motif in both pharmaceuticals and agrochemicals (Küçükgüzel and Şenkardeş, 2015; Khan et al., 2016). Pyrazole derivatives exhibit a wide range of biological activities including inflammatory (Bekhit and Abdel-Aziem, 2004), anticancer (Ma et al., 2013), and analgesic (Penning et al., 1997), as well as antifungal. For example, the 1,5-diaryl-3-trifluoromethylpyrazole motif of celecoxib has been used for novel antifungal pesticide development (Ma et al., 2020). More relevantly, we developed a series of pyrazole-bearing antifungal agrochemicals based on the scaffold of p21-activated protein kinase inhibitors, which demonstrated broad-spectrum and a high level of activity against various tested phytopathogenic fungi (Ma et al., 2018). In our attempt to search for effective fungicides for stem rot disease control, it would be interesting to study whether this unique 1,5-diaryl-3-formate platform could be exploited to identify potential antifungal candidates. In this study, we first characterized the antifungal activity of lead compound **3c** against the growth of *S. rolfsii*, which indeed exhibited clear, though modest, suppressive effect against the growth of the fungal pathogen. The following structural modification of lead compound focused on introducing various substituents on the phenyl ring attached to the N1 atom of the pyrazole ring, giving rise to a focused library of 15 compounds. Screening this library identified compound **6** with obviously increased activity. Finally, compound **6** was found rivaling the positive control in management of stem rot disease in field study.

## Materials and Methods

### Instrumentation and Chemicals

All chemical reagents were purchased without further purification before use and flash column chromatography was performed over silica gel 200–300 mesh. Analytical thin-layer chromatography (TLC) was performed with silica gel plates using silica gel 60 GF254 (Qingdao Haiyang Chemical Co., Ltd.). Melting points were determined with an X-4 melting points apparatus (Shanghai) and were uncorrected. ^1^H NMR and ^13^C NMR spectra were recorded on a Bruker AM-400 spectrometer at 400 and 100 MHz, respectively. Chemical shifts are reported in parts per million relative to internal tetramethylsilane (TMS). All the tested compounds hold a purity of at least 95%. Analytical high performance liquid chromatography (HPLC) was performed on the Thermo Ultimate 3000 equipped with Thermo Scientific Acclaim-C18 column and UV wavelength set at 254 nm. Eluent: 40% MeOH in H_2_O; flow rate = 1 ml/min.

### General Procedure for Methyl 1,5-Aryl-1H-Pyrazole-3-Carboxylate (1–15)

A mixture of 1,3-dione adducts (0.05 mol) with selected substituted arylhydrazines (0.06 mol) in ethanol or N,N-dimethylformamide (DMF) (200 ml) was refluxed for 8 h. Water was then added after the reaction mixture was cooled down to room temperature. The resulting precipitate was collected and recrystallized from methanol to give target compounds.

*methyl 5-(4-fluorophenyl)-1-(4-(methylsulfonyl)phenyl)-1H-pyrazole-3-carboxylate (1).*

Yield = 65%; white solid; mp: 78–79°C; ^1^H NMR (400 MHz, DMSO-*d*_6_) δ: 7.99 (d, J = 8.4 Hz, 2H), 7.58 (d, J = 8.4 Hz, 2H), 7.37 (dd, J = 8.8 Hz, 1.6 Hz, 2H), 7.27 (t, J = 8.8 Hz, 2H), 7.19 (s, 1H), 3.87 (s, 3H), 3.27 (s, 3H); ^13^C NMR (100 MHz, DMSO-*d*_6_) δ: 162.8 (d, JCF = 246 Hz), 162.3, 144.5, 144.3, 143.1, 140.9, 131.6 (d, JCF = 8.6 Hz, 2C), 128.6 (2C), 126.5 (2C), 125.5 (d, JCF = 3.1 Hz), 116.4 (d, JCF = 21.8 Hz, 2C), 110.9, 52.4, 43 7; HRMS (m/z) [M+Na]^+^ cacld. for C18H15FN2O4S 397.0634, found 397.0625.

*methyl 5-(4-fluorophenyl)-1-(2-nitrophenyl)-1H-pyrazole-3-carboxylate (2).*

Yield = 50%; brown solid; ^1^H NMR (400 MHz, DMSO-*d*_6_) δ: 8.15 (dd, J = 8.0 Hz, 1.6 Hz, 1H), 7.84 (td, J = 8.0 Hz, 1.6 Hz, 1H), 7.79 (td, J = 8.0 Hz, 1.6 Hz, 1H), 7.62 (dd, J = 8.0 Hz, 1.6 Hz, 1H), 7.32 (dd, J = 8.8 Hz, 5.6 Hz, 2H), 7.22 (t, J = 8.8 Hz, 2H), 7.22 (s, 1H), 3.85 (s, 3H); ^13^C NMR (100 MHz, DMSO-*d*_6_) δ: 162.8 (d, JCF = 246.3 Hz), 162.0, 145.8, 145.1, 144.7, 134.9, 132.2, 131.7, 131.2 (d, JCF = 8.7 Hz, 2C), 130.3, 126.1, 124.7 (d, JCF = 3.3 Hz), 116.3 (d, JCF = 21.8 Hz, 2C), 109.6, 52.4. HRMS (m/z) [M+Na]^+^ cacld. for C17H12FN3O4 364.0710, found 364.0701.

*methyl 1-(4-bromophenyl)-5-(4-fluorophenyl)-1H-pyrazole-3-carboxylate (3).*

Yield = 54%; white solid; mp: 107–109°C; ^1^H NMR (400 MHz, DMSO-*d*_6_) δ: 7.67 (d, J = 8.8 Hz, 2H), 7.34 (dd, J = 8.8 Hz, 5.6 Hz, 2H), 7.29 (d, J = 8.8 Hz, 2H), 7.25 (t, J = 8.8 Hz, 2H), 7.16 (s, H), 3.86 (s, 3H); ^13^C NMR (100 MHz, DMSO-*d*_6_) δ: 162.7 (d, JCF = 246 Hz), 162.3, 143.9, 143.9, 138.6, 132.7 (2C), 131.5 (d, JCF = 8.6 Hz, 2C), 127.9 (2C), 125.6 (d, JCF = 3.2 Hz), 122.1, 116.3 (d, JCF = 21.8 Hz, 2C), 110.4, 52.3. HRMS (m/z) [M+Na]^+^ cacld. for C17H12BrFN2O2 375.0144, found 375.0133.

*methyl 1-(2,4-dichlorophenyl)-5-(4-fluorophenyl)-1H-pyrazole-3-carboxylate (4).*

Yield = 70%; white solid; mp: 94–96°C; ^1^H NMR (400 MHz, DMSO-*d*_6_) δ: 7.86 (d, J = 2.4 Hz, 1H), 7.79 (d, J = 8.4 Hz, 1H), 7.65 (dd, J = 8.4 Hz, 2.4 Hz, 1H), 7.31 (dd, J = 8.8 Hz, 5.6 Hz, 2H), 7.22 (s, 1H), 7.21 (t, J = 8.8 Hz, 2H), 3.86 (s, 3H); ^13^C NMR (100 MHz, DMSO-*d*_6_) δ: 162.7 (d, JCF = 245.9 Hz), 162.2, 145.6, 144.4, 136.2, 135.9, 132.3, 132.0, 130.7 (d, JCF = 8.6 Hz, 2C), 130.3, 129.1, 125.2 (d, JCF = 3.2 Hz), 116.3 (d, JCF = 21.8 Hz, 2C), 108.9, 52.3; HRMS (m/z) [M+Na]^+^ cacld. for C17H11Cl2FN2O2 387.0079, found 387.0072.

*methyl 1,5-bis(4-fluorophenyl)-1H-pyrazole-3-carboxylate (5).*

Yield = 65%; yellow solid; mp: 92–94°C; ^1^H NMR (400 MHz, DMSO-*d*_6_) δ: 7.98 (dd, J = 8.8 Hz, 5.6 Hz, 2H), 7.64 (s, 1H), 7.61 (dd, J = 8.8 Hz, 4.8 Hz, 2H), 7.35 (t, J = 8.8 Hz, 2H), 7.28 (t, J = 8.8 Hz, 2H), 3.77 (s, 3H); ^13^C NMR (100 MHz, DMSO-*d*_6_) δ: 162.7 (d, JCF = 246 Hz), 162.1 (d, JCF = 246 Hz), 159.2, 150.2, 136.7 (d, JCF = 2.9 Hz), 134.8, 128.6 (d, JCF = 9.0 Hz, 2C), 128.6 (d, JCF = 3.2 Hz), 128.0 (d, JCF = 8.3 Hz, 2C), 116.3 (d, JCF = 21.5 Hz, 2C), 115.9 (d, JCF = 22.9 Hz, 2C), 109.8, 52.6. HRMS (m/z) [M+Na]^+^ cacld. for C17H12F2N2O2 315.0945, found 315.0952.

*methyl 1-(3-chloropyridin-2-yl)-5-(4-fluorophenyl)-1H-pyrazole-3-carboxylate(6).*

Yield = 80%; yellow solid; mp: 98–99°C; ^1^H NMR (400 MHz, DMSO-*d*_6_) δ: 7.54–7.50 (m, 2H), 7.46 (t, J = 8.8 Hz, 1H), 7.35 (dd, J = 8.8 Hz, 5.6 Hz, 2H), 7.25 (t, J = 8.8 Hz, 2H), 7.25 (s, 1H), 3.86(s, 3H); ^13^C NMR (100 MHz, DMSO-*d*_6_) δ: 162.6 (d, JCF = 245.7 Hz), 162.1, 143.9, 143.8, 140.4, 133.7, 131.4 (d, JCF = 8.6 Hz, 2C), 131.1, 129.0, 125.8, 125.4 (d, JCF = 3.3 Hz), 124.6, 116.2 (d, JCF = 21.8 Hz, 2C), 110.2, 52.4; HRMS (m/z) [M+Na]+ cacld. for C16H11CIFN3O2 354.0422, found 354.0432.

*methyl 1-(2-methyl)-5-(4-fluorophenyl)-1H-pyrazole-3-carboxylate (7).*

Yield = 85%; yellow solid; mp: 92–93°C; ^1^H NMR (400 MHz, DMSO-*d*_6_) δ: 7.42 (dt, J = 8.0 Hz, 1.6 Hz, 1H), 7.37–7.34 (m, 3H), 7.26 (dd, J = 8.8 Hz, 5.6 Hz, 2H), 7.21 (s, 1H), 7.16 (t, J = 8.8 Hz, 2H), 3.85 (s, 3H), 1.86 (s, 3H); ^13^C NMR (100 MHz, DMSO-*d*_6_) δ: 162.5 (d, JCF = 245.8 Hz), 162.4, 144.7, 143.5, 138.8, 135.1, 131.4, 130.5 (d, JCF = 8.5 Hz, 2C), 130.3, 128.3, 127.4, 125.6 (d, JCF = 3.2 Hz), 116.2 (d, JCF = 21.7 Hz, 2C), 108.8, 52.2, 17.2; HRMS (m/z) [M+Na]+ cacld. for C18H15FN2O2 333.1015, found 333.1005.

*methyl 1-(4-chlorophenyl)-5-(4-fluorophenyl)-1H-pyrazole-3-carboxylate (8).*

Yield = 74%; yellow solid; mp: 77–78°C; ^1^H NMR (400 MHz, DMSO-*d*_6_) δ: 7.55 (d, J = 8.8 Hz, 2H), 7.37 (d, J = 8.8 Hz, 2H), 7.34 (dd, J = 8.0 Hz, 1.6 Hz, 2H), 7.25 (t, J = 8.8 Hz, 2H), 7.16 (s, 1H), 3.86 (s, 3H); ^13^C NMR (100 MHz, DMSO-*d*_6_) δ: 162.7 (d, JCF = 246 Hz), 162.3, 143.9, 143.8, 138.2, 133.6, 131.5 (d, JCF = 8.6 Hz, 2C), 129.8 (2C),127.8 (2C), 125.6 (d, JCF = 3.1 Hz), 116.3 (d, JCF = 21.8 Hz, 2C), 110.3, 52.3; HRMS (m/z) [M+Na]+ cacld. for C17H12CIFN2O2 353.0469, found 353.0463.

*methyl 5-(4-fluorophenyl)-1-(4-(trifluoromethyl)phenyl)-1H-pyrazole-3-carboxylate (9).*

Yield = 74%; brown solid; mp: 82–83°C; ^1^H NMR (400 MHz, DMSO-*d*_6_) δ: 7.86 (d, J = 8.4 Hz, 2H), 7.57 (d, J = 8.0 Hz, 2H), 7.36 (dd, J = 8.8 Hz, 5.6 Hz, 2H), 7.26 (t, J = 8.8 Hz, 2H), 7.19 (s, 1H), 3.87 (s, 3H); ^13^C NMR (100 MHz, DMSO-*d*_6_) δ: 162.8 (d, JCF = 246 Hz), 162.3, 144.4, 144.2, 142.5 (d, JCF = 5.6 Hz), 131.7 (d, JCF = 8.6 Hz, 2C), 129.1 (q, JCF = 32 Hz), 126.9 (q, JCF = 3.5 Hz), 126.5 (4C), 125.6 (d, JCF = 3.2 Hz), 116.3 (d, JCF = 21.9 Hz, 2C), 110.8, 52.3; HRMS (m/z) [M+Na]+ cacld. for C18H12F4N2O2 387.0733, found 387.0727.

*methyl 1-(3-chlorophenyl)-5-(4-fluorophenyl)-1H-pyrazole-3-carboxylate(* ***10*** *).*

Yield = 83%; brown solid; mp: 106–108°C; ^1^H NMR (400 MHz, DMSO-*d*_6_) δ: 7.54 (m, 2H), 7.46 (t J = 8.0 Hz, 2H), 7.35 (dd, J = 8.4 Hz, 5.6 Hz, 2H), 7.25 (t, J = 8.8 Hz, 2H), 7.25 (s, 1H), 7.16 (s, 1H), 3.87 (s, 3H); ^13^C NMR (100 MHz, DMSO-*d*_6_) δ: 162.6(d, JCF = 245.7 HZ), 162.1, 143.9, 143.8, 140.4, 133.7, 131.4 (d, JCF = 8.6 Hz, 2C), 131.1, 129.0, 125.8, 125.4 (d, JCF = 3.3 Hz), 124.6, 116.2 (d, JCF = 21.8 Hz, 2C), 110.2, 52.2; HRMS (m/z) [M+Na]+ cacld. for C17H12CIFN2O2 353.0469, found 353.0463.

*methyl 1-(2,6-dichlorophenyl)-5-(4-fluorophenyl)-1H-pyrazole-3-carboxylate (11).*

Yield = 80%; white solid; mp: 94–96°C; ^1^H NMR (400 MHz, DMSO-d6) δ: 7.71 (d, J = 5.6 Hz, 1H), 7.69 (s, 1H), 7.62 (dd, J = 9.2 Hz, 7.2 Hz, 1H), 7.28 (dd, J = 8.8 Hz, 5.6 Hz, 2H), 7.26 (s, 1H), 7.21 (t, J = 8.8 Hz, 2H), 3.84 (s, 3H), ^13^C NMR (100 MHz, DMSO-*d*_6_) δ: 162.9 (d, JCF = 246.4 Hz), 162.1, 145.8, 145.1, 134.8, 134.0 (2C), 133.4, 130.1 (d, JCF = 8.7 Hz, 2C), 129.8 (2C), 124.9 (d, JCF = 3.3 Hz), 116.5 (d, JCF = 21.9 Hz, 2C), 108.9, 52.5; HRMS (m/z) [M+Na]+ cacld. for C17H11CI2FN2O2 387.0079, found 387.0073.

*methyl 5-(4-fluorophenyl)-1-(4-methoxyphenyl)-1H-pyrazole-3-carboxylate* (***12***).

Yield = 80%; white crystal; mp: 219–221°C; ^1^H NMR (400 MHz, DMSO-*d*_6_) δ: 7.31 (dd, J = 8.8 Hz, 5.6 Hz, 2H), 7.25 (d, J = 8.8 Hz, 2H), 7.21 (t, J = 8.8 Hz, 2H), 7.12 (s, 1H), 6.99 (d, J = 8.8 Hz, 2H), 3.85 (s, 3H), 3.79 (s, 3H); ^13^C NMR (100 MHz, DMSO-*d*_6_) δ: 162.5 (d, JCF = 246 Hz), 162.5, 159.6, 143.8, 143.2, 132.5, 131.3 (d, JCF = 8.5 Hz, 2C), 127.5 (2C), 125.9 (d, JCF = 3.3 Hz), 116.3 (d, JCF = 21.7 Hz, 2C), 114.8 (2C), 109.7, 55.9, 52.2; HRMS (m/z) [M+Na]+ cacld. for C18H15FN2O3 349.0964, found 349.0957.

*methyl 5-(4-fluorophenyl)-1-(4-(trifluoromethoxy)phenyl)-1H-pyrazole-3-carboxylate* ***(13)***.

Yield = 60%; white crystal; mp: 114–115°C; ^1^H NMR (400 MHz, DMSO-*d*_6_) δ: 7.46 (s, 4H), 7.33 (dd, J = 8.8 Hz, 5.6 Hz, 2H), 7.22 (t, J = 8.8 Hz, 2H), 7.14 (s, 1H), 3.85 (s, 3H), ^13^C NMR (100 MHz, DMSO-*d*_6_) δ: 162.7 (d, JCF = 245.7 Hz), 162.2, 148.2(d, JCF = 1.8 Hz, 143.9, 143.8, 138.3, 131.5 (d, JCF = 8.6 Hz, 2C), 128.0 (2C), 125.6 (d, JCF = 3.1 Hz), 122.2 (2C), 120.3 (q, JCF = 255.8 Hz), 116.3 (d, JCF = 21.9 Hz, 2C), 110.4, 52.3; HRMS (m/z) [M+Na]+ cacld. for C18H12F4N2O3 403.0682, found 403.0674.

*methyl 1-(4-cyano-2-fluorophenyl)-5-(4-fluorophenyl)-1H-pyrazole-3-carboxylate* (***14***).

Yield = 55%; yellow liquid; ^1^H NMR (400 MHz, DMSO-*d*_6_) δ: 8.09–8.07 (m, 1H), 7.91–7.90 (m, 2H), 7.24 (dd, J = 8.4 Hz, 5.6 Hz, 2H), 7.23 (s, 1H), 7.20 (t, J = 8.4 Hz, 2H), 3.85 (s, 3H); ^13^C NMR (100 MHz, DMSO-*d*_6_) δ: 162.7 (d, JCF = 246 Hz), 162.0, 155.4 (d, JCF = 252 Hz), 145.8, 145.2, 131.3 (d, JCF = 11.7 Hz), 131.0, 130.7 (d, JCF = 8.7 Hz, 2C), 130.4 (d, JCF = 8.6 Hz), 125.1 (d, JCF = 3.1 Hz), 121.6 (d, JCF = 23.6 Hz), 117.3 (d, JCF = 2.4 Hz), 116.3 (d, JCF = 21.9 Hz, 2C), 114.5 (d, JCF = 9.5 Hz), 109.6, 52.3; HRMS (m/z) [M+Na]+ cacld. for C18H11F2N3O2 362.0717, found 362.0708.

*4-(5-(4-fluorophenyl)-3-(methoxycarbonyl)-1H-pyrazol-1-yl)benzoic acid* (***15***).

Yield = 55%; white solid; mp: 51–53°C; ^1^H NMR (400 MHz, DMSO-*d*_6_) δ: 8.00 (d, J = 8.8 Hz, 2H), 7.44 (d, J = 8.8 Hz, 2H), 7.34 (dd, J = 5.6 Hz, 3.2 Hz, 2H), 7.24 (t, J = 8.8 Hz, 2H), 7.18 (s, 1H), 3.87 (s, 3H), ^13^C NMR (100 MHz, DMSO-*d*_6_) δ: 166.8, 162.3 (d, JCF = 246 Hz), 161.5, 144.1, 144.0, 142.6, 131.5 (d, JCF = 8.8 Hz, 2C), 131.0, 130.7 (2C), 125.8 (2C), 125.6 (d, JCF = 3.2 Hz), 116.3 (d, JCF = 22.9 Hz, 2C), 110.6, 52.3; HRMS (m/z) [M-H]^+^ cacld. for C18H13FN2O4 339.0781, found 339.0794.

### Antifungal Assays

The pathogenic isolate was isolated from Kaifeng, Hennan Province, China PRC, where farmers usually use triazole fungicides such as tebuconazole or difenoconazole for both seed coating and foliar spray to control crop disease. The DNA sequencing results of the pathogen isolate were deposited in GenBank under accessions number OK275413. The antifungal activity of newly synthesized compounds was tested following a modification of a previously described protocol (Zeun et al., 2013). Briefly, to 1,000 ml of double-distilled water was added 40 g of potato dextrose agar (PDA) powder, which was boiled and mixed to dissolve. The medium prepared was autoclaved at 120°C for 20 min before cooled down to 55°C. The compounds stock solutions in DMSO diluted by mixing with the medium to give the final indicated concentrations that were poured into Petri dishes (9 cm diameter). A treatment with vehicle (DMSO) was included as a reference, and three replicates were performed for each treatment. Each Petri dish was inoculated with a mycelia disc (1 mm diameter) pin-picked from the margin of freshly prepared source colonies of the respective fungus growing on a PDA medium. After incubation in the dark for 72 h, all three diameters of the mycelium for each treatment were measured, averaged, and converted to percentage activity relative to vehicle treatment. The four-parameter logistic regression model was used for analysis of the dose-response inhibition of compound **6**, tebuconazole, and thifluzamine as well as calculating their respective IC_50_. The regression curves were generated by using the R package (version 3.3.3).

### Field Study

A field-plot study was conducted at an experimental station of Henan Academy of Agricultural Sciences in North Henan Province, on the north bank of the Yellow River in 2018 and 2019. The soil type was loamy fine sand. To guarantee the inoculum in the test area, *S*. *rolfsii* was cultured on autoclaved peanut shell, dried and uniformly scattered over the plots 4 weeks after planting peanuts. The experimental design was a randomized complete block with treatment replicated four times. All plots had four rows 6 m long spaced on 80-cm centers. Data were taken from the two middle rows of each 4-row subplot to minimize the spray drift effect from adjacent plots. Fungicides were evaluated as foliar sprays for control of southern stem rot. Postemergence herbicide quizalofop-p-ethyl (60 g a.i./ha Henanlvbao Co., Ltd.) was applied about 40 days after planting. Chlorothalonil (0.8 kg a.i./ha, Anyangruipu Co., Ltd.) was used for leafspot management as it has no significant field activity on *S*. *rolfsii*. The sprays for compound **6**, thifluzamide, and tebuconazole were applied twice, at about 6 and 8 weeks after planting, with a compressed-air pressurized knapsack electric sprayer which delivered 225 l/ha at about 200 kPa using three conventional solid full cone brass nozzles per boom row. Southern stem rot disease loci were counted 2 weeks prior to digging (one locus was defined as no more than 30 cm of consecutive southern stem rot damaged plants in a row). Peanuts were harvested and yields were reported at 10% moisture. Significance of treatment effects was tested by analysis of variance and Fisher’s protected least significant difference (LSD) test (*P* = 0.05).

## Results and Discussion

### Chemistry

**SCHEME 1:**
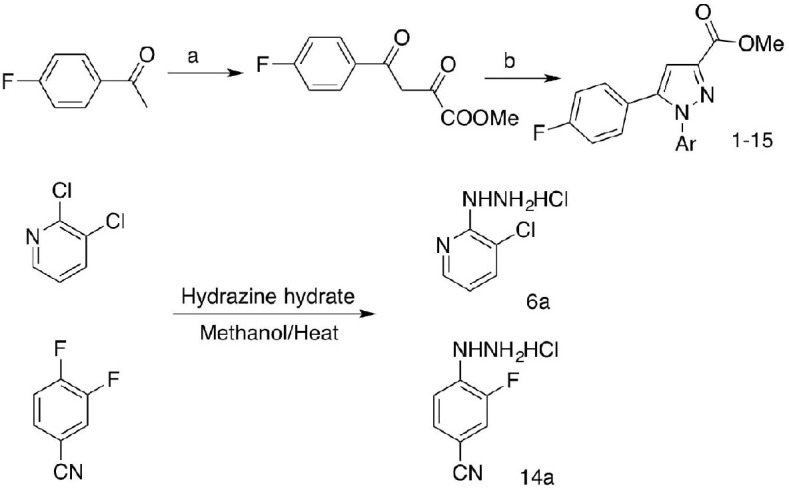
Synthetic route to compounds **1–15**. Reagents and conditions: (a) diethyl oxalate, NaOMe/methanol; (b) substituted arylhydrazine-HCl, DMF, 60–70°C.

The compound library was established following Scheme 1 as previously reported (Ma et al., 2018, 2020). Briefly, 4-fluoroacetophenone was condensed with diethyl oxalate in methanol to generate 1,3-diketone methyl ester instead of ethyl ester for the ester exchange process in the presence of sodium methoxide. Target compounds were furnished by cyclizing the 1,3-diketone with various aromatic hydrazines either commercially available or prepared by nucleophilic aromatic substitution of corresponding aromatic halides with hydrazine hydrate in methanol (**6a, 14a)**.

### Antifungal Potential of Lead Compound **3c**

**FIGURE 1:**
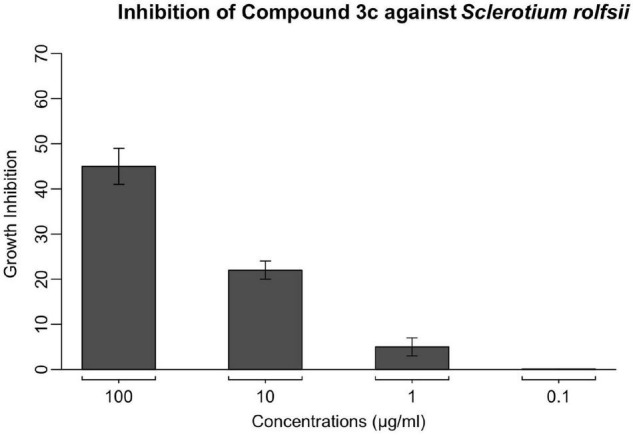
The inhibitory effect of lead compound **3c** against *S. rolfsii* was measured using PDA assay at concentrations of 0.1, 1, 10, and 100 μg/ml. Points indicate means, and bars indicate SD (*n* = 4).

To evaluate its inhibitory effect against the growth of *S. rolfsii*, the lead compound **3c** cross a wide range of concentrations was examined on PDA medium and the inhibition rate is illustrated in Figure 1. As shown, compound **3c** did not exhibit obvious suppression on the mycelia growth at concentrations less than 10 μg/ml. Specifically, around 22% inhibition rate was observed at 10 μg/ml, and this growth suppression was elevated to 45% as the concentration of compound **3c** was brought to 100 μg/ml. This formed a sharp contrast with the lead compound’s complete inhibition against those susceptible fungal pathogens reported in our previous study (Ma et al., 2018), and *S. rolfsii* seems more tolerant to compound **3c**, indicating the species-dependence of compound **3c**’s antifungal effect. On the other hand, even though compound **3c** demonstrated relatively moderate inhibition on the growth of *S. rolfsii*, the clear suppressive effect encouraged us to explore its potential of acting as a lead for more potent antifungal development through structural decoration of the 1,5-diaryl-pyrazole-3-formate scaffold.

### Structure and Antifungal Activity Relationship

A focused library of 15 derivatives was established primarily by structurally modifying the aromatic ring attached to the N1 atom of the lead compound. Specifically, various arylhydrazine hydrochloride either commercially available or obtained through one-step substitution transformation with hydrazine hydrate were selected and cyclized with diketones to furnish the final compounds, whose inhibitory potency was examined at 100 μg/ml and the results are listed in Table 1. Generally, most of the analogs did not demonstrate notable inhibition effect over 30% except for compounds **6**, **8**, and **15**. Among these three candidates, compounds **8** and **15** showed 38 and 32% inhibition against the growth of *S. rolfsii*, respectively. On the contrary, compound **6** exhibited almost twofold improved activity as evidenced by its nearly 90% inhibition compared with lead compound’s inhibition of lower than 50%.

**TABLE 1:** Inhibition rate of compound **3c** and its derivatives against *S. rolfsii* at 100 μg/ml.

		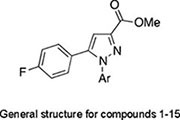			

Compound	Ar	% Growth inhibition	Compound	Ar	% Growth inhibition
Lead	4-CN-C_6_H_4_-	45%	9	4-CF_3_-C_6_H_4_-	14%
1	4-SO_2_Me-C_6_H_4_-	24%	10	3-Cl-C_6_H_4_-	10%
2	2-NO_2_-C_6_H_4_-	24%	11	2,6-dichlorophenyl	13%
3	4-Br-C_6_H_4_-	19%	12	4-OMe-C_6_H_4_-	0%
4	2,4-dichlorophenyl	19%	13	4-OCF_3_-C_6_H_4_-	5%
5	4-F-C_6_H_4_-	14%	14	2-F-4-CN-C_6_H_3_-	28%
6	3-chloropyridinyl	92%	15	4-COOH-C_6_H_4_-	32%
7	2-Me-C_6_H_4_-	27%	Tebuconazole		100%
8	4-Cl-C_6_H_4_-	38%	Thifluzamide		100%

*General structure for compounds **1–15**.*

**FIGURE 2:**
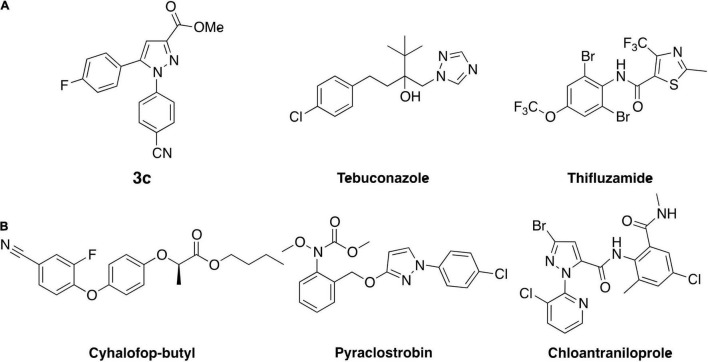
**(A)** Structure of lead compound **3c** and fungicide controls tebuconazole as well as thifluzamide. **(B)** Commercial pesticides whose synthetic materials were used to derive compound **3c**.

In addition, compounds **6**, **8**, and **14** were designed and prepared because the hydrazine hydrochloride salts used to synthesize these three compounds are also the key intermediates to prepare pyraclostrobin, cyhalofop-butyl, and chloantraniloprole, respectively (Figure 2), which are widely used fungicide, herbicide, or insecticide consumed in the scale of millions of tons annually. These commodity chemicals are supposed to be available in large quantities at a reasonable price, and thus would facilitate the commercialization process of these novel antifungals under development. However, compound **8** showed slightly decreased activity over parent compounds even though the 4-chlorophenylpyrazole moiety is also present in the well-known fungicidal pesticide pyraclostrobin (Figure 2). This is understandable concerning the supporting role of para-chlorphenylpyrazole to pyraclostrobin’s antifungal pharmacophore of methyl methoxycarbamate (equivalent to methyl beta-acrylamide of QoI fungicides). Even worse, adding one more –F atom to the lead compound to give compound **14** greatly dragged down the activity even though the fluorine atom is comparable to hydrogen in size and would not generate extra steric restriction upon binding with targets. The reasonable explanation for this dramatic decrease in activity might be that the extra fluorine atom changed the electron distribution circulating the aromatic ring. This scenario indicated the sensitivity of the antifungal activity of this series of compounds to the alteration of electronic effect. Compound **6** that has the 3-chloropyridinylpyrazole in its structure, demonstrated prominent activity against *S. rolfsii* as treatment at 100 μg/ml inhibited the fungus’ growth of by more than 90%. This was expected as the pyridinyl is bioisosteric to cyanophenyl in molecular design in medicinal chemistry. In addition, the presence of the pyridinyl group would deliver favorable physical properties such as elevated solubility, which may be able to increase the absorption and redistribution within plant. Moreover, in contrast to its role of primarily regulating the physiochemical properties of the widely used insecticide chloantrilaniphore, 3-chloropyrindylpyrazole in compound **6** was believed to constitute a wedge-like pharmacophore which would facilitate the entry of this series of novel antifungal chemicals into their binding pocket in target. Moreover, compound **6** also demonstrated antifungal activity against other pythopathogenic fungi with the inhibition rates ranging 42–78% (Supplementary Table 1).

**FIGURE 3:**
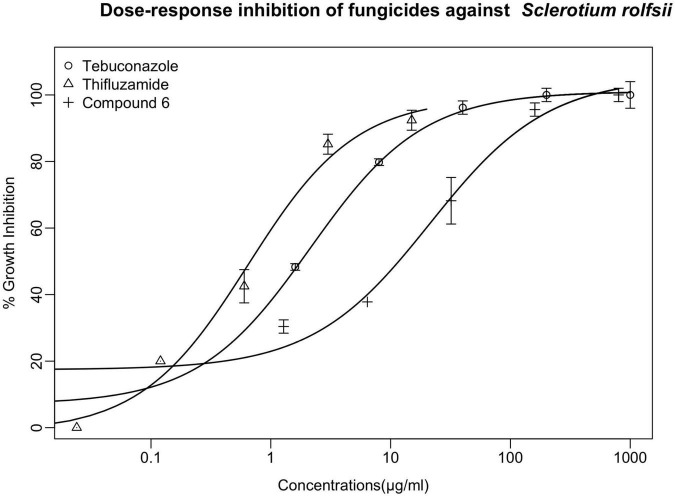
The dose-response inhibitory effects of compounds **6** and two controls (tebuconazole and thifluzamide) against *S. rolfsii* were measured with PDA assay. Points indicate means, and bars indicate SD (*n* = 4).

As the most active member in the series, compound **6** manifested slightly less antifungal effect than the two controls tebuconazole and thifluzamide, either of which was able to completely inhibit the growth of *S. rolfsii* at 100 μg/ml. To further compare the antifungal potency of compound **6** with the two controls, dose-response assay of these three chemicals against the tested fungus was conducted and the results are listed in Figure 3. As shown, the inhibitory activities were increased with the increment of tested concentrations. In addition, among the agents, thifluzamide was apparently the most potent with an IC_50_ of less than 1 μg/ml, followed by tebuconazole, which inhibited 50% of growth at a concentration of 1.8 μg/ml, higher than that of 0.08 μg/ml reported by Brenneman et al. (1991) Compound **6** was relatively less active and its IC_50_ was calculated to be 12 μg/ml, indicating that there were lots to play for structural modification to further elevate the activity of these series of compounds.

### Field Trials

Despite an order of magnitude difference in the activity between compound **6** and the controls, compound **6**’s control efficacy against peanut stem rot disease in the field was investigated as well concerning its relative different pharmacophore. Thifluzamide and tebuconazole, as the effective agents, were selected as positive controls. These two agents have been extensively used in China for stem rot control and are considered as first-line fungicidal pesticides by peanut growers for their relative better controlling efficacy compared with other registered commercial fungicides. The regular recommended application rate of thifluzamide and tebuconazole against peanut stem rot are approximately 72–144 and 60–120 g of active ingredients per hectare (g a.i.ha^–1^), respectively. For ease of comparison, the application rate of 100 g a.i.ha^–1^ was selected for the study.

Compound **6** in the formulation of 10% suspension concentrates (SC) in water as well as thifluzamide (240 g/L SC) and tebuconazole (430 g/L SC) were applied twice on a 14-day schedule at the end of June and early July in the year 2018 and 2019 as a full canopy spray. The results are listed in Table 2. In both trials, disease loci were significantly reduced with fungicide treatments (50–54%) compared with the untreated control. In 2018, compound **6** was similarly effective in controlling the disease to the two control fungicide treatments. Specifically, tebuconazole reduced the disease development by 54%, while thifluzamide and compound **6** treatment approximately resulted in a 50% decrease in disease incidence over the untreated control. Thifluzamide treatment and compound **6** in 2019 significantly reduced southern stem rot loci by 55 and 50% compared with the control, respectively, while the disease incidence was not significantly different between the two fungicide treatments.

**TABLE 2:** Effect of compound **6** and two controls on southern stem rot incidence and peanut yield in field study.

Treatment	Rate (kg ha^–1^)	Disease loci per plot^w^		Disease incidence^w^		Peanut yield (kg ha^–1^)^w^	
		2018	2019	2018	2019	2018	2019
Tebuconazole^v^	0.1	9.3 b	/	10.3% b	/	2430 a	/
Thifluzamide	0.1	10.3 b	13 b	11.4% b	14.4% b	2560 a	2010 a
Compound **6**	0.1	10 b	14 b	11.1% b	15.5% b	2355 a	2130 a
Control (water)	0.1	20.3 a	28.8 a	22.5% a	32.0% a	1950 b	1800 b

*^w^Means within a column followed by different letters are significantly different according to Fisher’s protected least significant difference (P = 0.05).*

*^v^Field study data for tebuconazole in 2019 was not collected.*

Pod yield responses were significantly higher for fungicide treatments over the untreated control for both trials. In 2018, fungicide treatments similarly resulted in significant increases in pod yields by at least 20% over the untreated control. In the year 2019, the pod yields for compound **6** and thifluzamide were also significantly higher over the untreated control. However, the difference in pod yield between these two fungicides was not significant, consistent with their similar levels of stem rot loci control.

Although the IC_50_ value of compound **6** against the pathogenic fungi of stem rot disease was greater than those of the controls, i.e., tebuconazole and thifluzamide, the field trial study showed compound **6** had comparable field control effect to the controls. Many factors especially fungicides’ systemicity may be the potential reasons for this apparent discrepancy (Augusto and Brenneman, 2012). As one of important soilborne diseases, peanut stem rot disease occurs in the surface of the soil. By the time of fungicide application, the peanut canopy has formed a thick layer of overlapping leaves, which makes it difficult for the fungicides typically applied over the top of the peanut canopy to penetrate the dense foliage to reach the stem rot (Augusto et al., 2010a,b). Beside the application techniques to help improve fungicide penetration and deposition on the soilborne pathogen such as modification of the sprayer to open the canopy or applying fungicides at night when the leaves are folded, systemic fungicides with basipetal movement to target soilborne pathogens by moving downward in peanut after foliage application is critical to maintain improved disease control or enhance pod yield. We thus examined the octanol-water partition coefficient (Kow) using the HPLC (Mirrlees et al., 1976) method for the fungicides, which is a critical measure of a chemical’s hydrophilicity/lipophilicity and determines distribution of a chemical in a living organism (Cumming and Rücker, 2017). The tested LogKow for compound **6** was 2.7, lower than those of 3.7 and 3.4 associated with tebuconazole and thifluzamide, respectively. The intermediate LogKow value of these fungicides would allow for their penetration through the cuticle of peanut leaves. More importantly, the lower LogKow value means more hydrophilic, indicating that compound **6** would be more soluble in aqueous environment within peanut than tebuconazole or thifluzamide. This warrants further thorough investigation.

In summary, this study described our effort to develop novel fungicides by lead optimization of our previously identified active antifungal chemical compound **3c**. Among the prepared analogs, compound **6** demonstrated prominent inhibitory effects against the growth of *S. rolfsii* with an IC_50_ of 12 μg/ml, more active than any other analogs in the series. This may relate to the presence of a pyridine ring in compound **6**, the only compound in the derivatives to contain a heterocycle connected to the N1 atom of the pyrazole skeleton. Heterocyclic moieties are frequently accompanied with higher activity. For example, this scenario was observed in the modification of chlorantraniliprole, where replacement of the pyridine subunit with many substituted phenyl groups just decreased the activity. Electron-negativity of the hetero atoms in the cyclic ring systems of heterocyclic compounds can cause unevenly distributed electron density in the π-molecular orbital compared with benzene ring, which would generate stronger interactions between small molecules and their protein targets. More importantly, hetero atoms (especially N atom) are able to form hydrogen bonds with specific functional groups in the targets, further strengthening their binding affinity. Therefore, introduction of heterocycles to make isosteric replacements is important for molecular design and would be adapted in our future drug development. In addition, field study suggested comparable controlling effect of compound **6** to the controls in terms of the number of disease loci, disease incidences, and peanut yields. The encouraging results indicate compound **6** may be a potential antifungal pesticide candidate against the stem rot disease. On the other hand, the relatively lower potency of compound **6** in directly inhibiting the growth of fungal pathogens indicated that there is much room for further elevating the potency of this class of compound through structural modification. The next generation of optimization would focus on the variation of the methyl ester moiety. This work is undergoing in our lab.

## Data Availability Statement

The original contributions presented in the study are included in the article/Supplementary Material, further inquiries can be directed to the corresponding author/s.

## Author Contributions

YM designed the experiment and wrote the manuscript. JY performed the compounds preparation. DY performed the PDA assay in the lab. GQ and JZ performed the field study investigation. All authors contributed to the article and approved the submitted version.

## Conflict of Interest

The authors declare that the research was conducted in the absence of any commercial or financial relationships that could be construed as a potential conflict of interest.

## Publisher’s Note

All claims expressed in this article are solely those of the authors and do not necessarily represent those of their affiliated organizations, or those of the publisher, the editors and the reviewers. Any product that may be evaluated in this article, or claim that may be made by its manufacturer, is not guaranteed or endorsed by the publisher.
