# Supplementary material for: Development of: 1,5-Diaryl-Pyrazole-3-Formate Analogs as Antifungal Pesticides and Their Application in Controlling Peanut Stem Rot Disease
Source: Front Microbiol. 2022 Jan 4;12:728173. doi: 10.3389/fmicb.2021.728173 (PMC8763808; doi:10.3389/fmicb.2021.728173)
Supplement: Supplementary file 1 [file Data_Sheet_1.PDF]

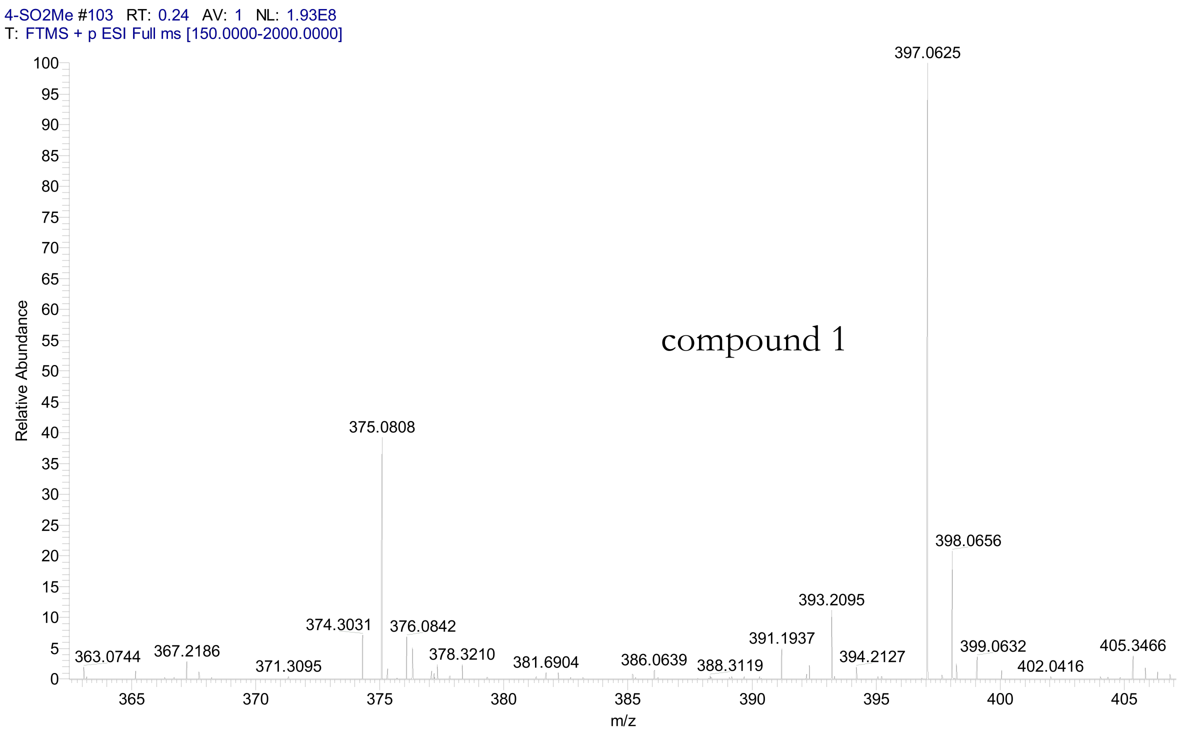
4-SO2Me #103 RT: 0.24 AV: 1 NL: 1.93E8
T: FTMS + p ESI Full ms [150.0000-2000.0000]
397.0625
398.0656
393.2095
375.0808
374.3031
376.0842
363.0744
367.2186
371.3095
378.3210
381.6904
386.0639
388.3119
391.1937
394.2127
399.0632
402.0416
405.3466
Relative Abundance
m/z
compound 1

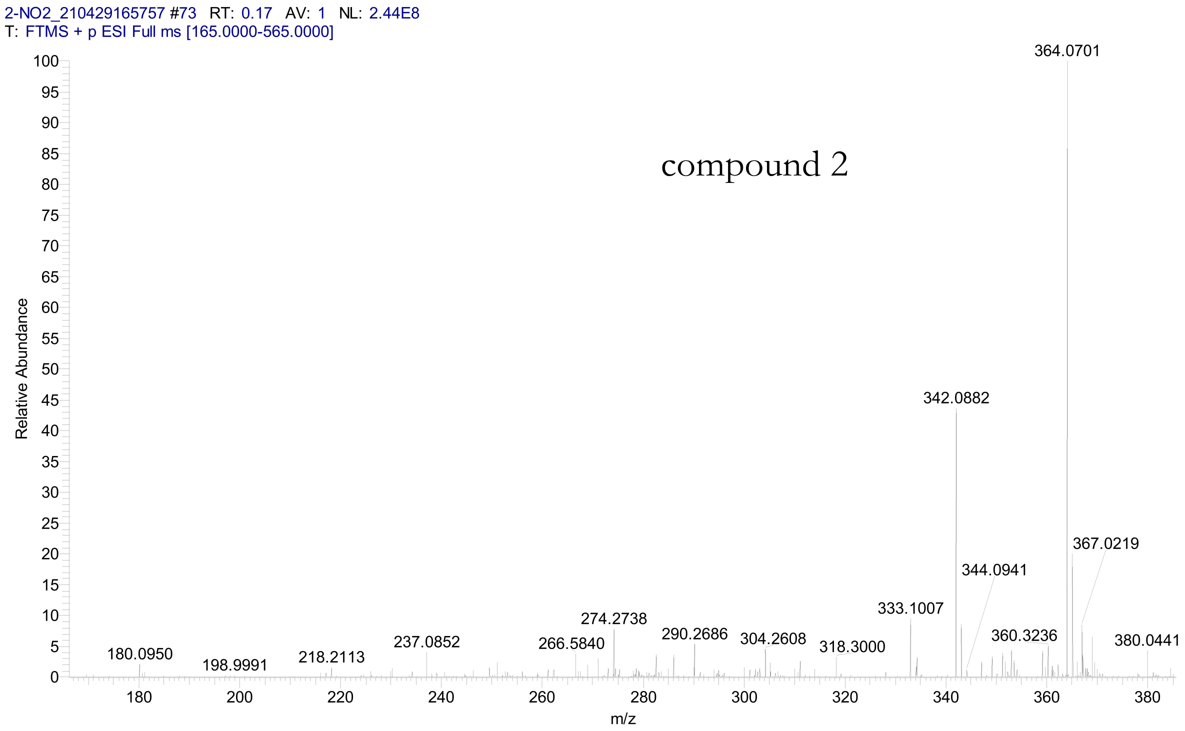

2-NO2_210429165757 #73 RT: 0.17 AV: 1 NL: 2.44E8
T: FTMS + p ESI Full ms [165.0000-565.0000]
compound 2
Relative Abundance
m/z
364.0701
342.0882
367.0219
344.0941
333.1007
274.2738
290.2686
304.2608
360.3236
380.0441
237.0852
266.5840
318.3000
180.0950
218.2113
198.9991

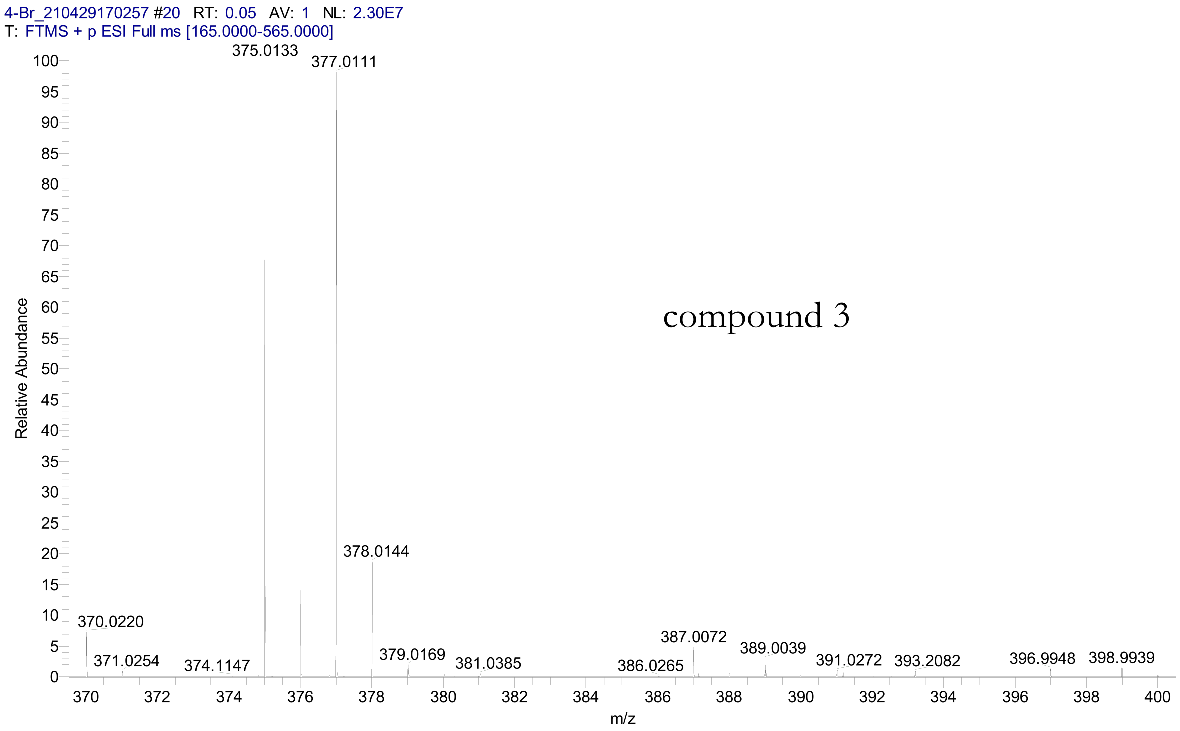
4-Br_210429170257 #20 RT: 0.05 AV: 1 NL: 2.30E7
T: FTMS + p ESI Full ms [165.0000-565.0000]
375.0133
377.0111
378.0144
370.0220
371.0254
374.1147
379.0169
381.0385
386.0265
387.0072
389.0039
391.0272
393.2082
396.9948
398.9939
Relative Abundance
m/z

compound 3

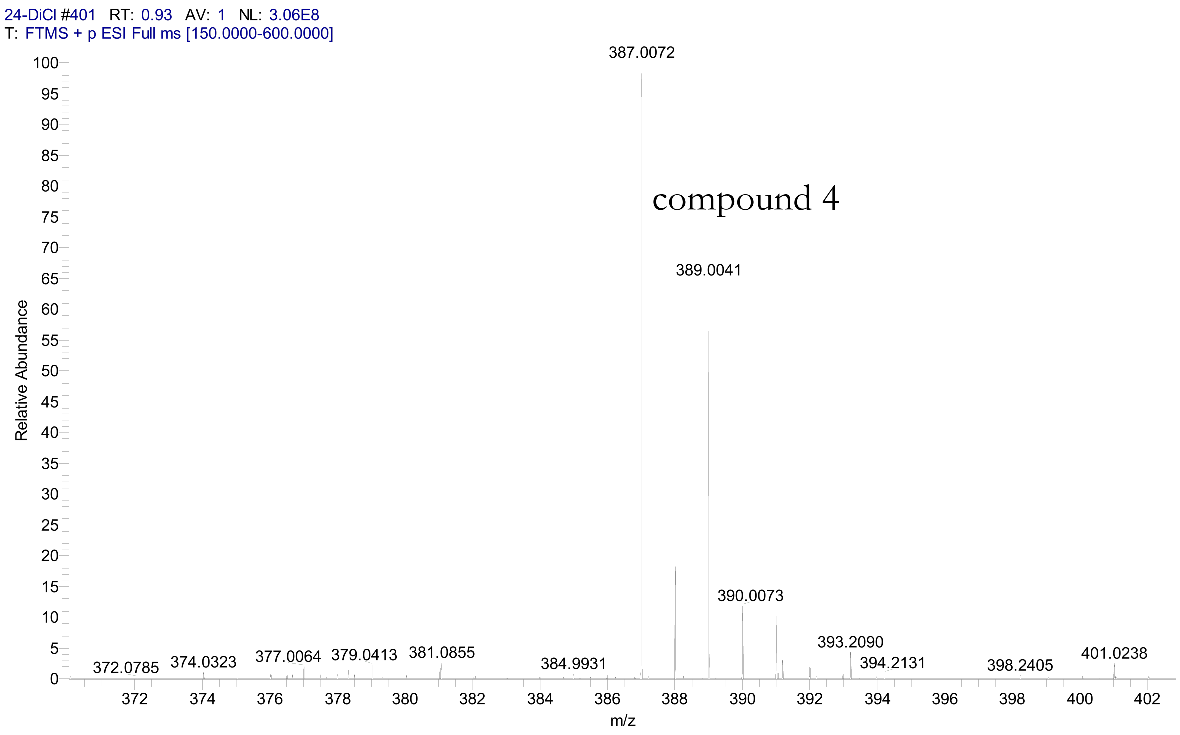
24-DiCl #401 RT: 0.93 AV: 1 NL: 3.06E8
T: FTMS + p ESI Full ms [150.0000-600.0000]
Relative Abundance
m/z
387.0072
389.0041
390.0073
393.2090
394.2131
398.2405
401.0238
372.0785
374.0323
377.0064
379.0413
381.0855
384.9931
compound 4

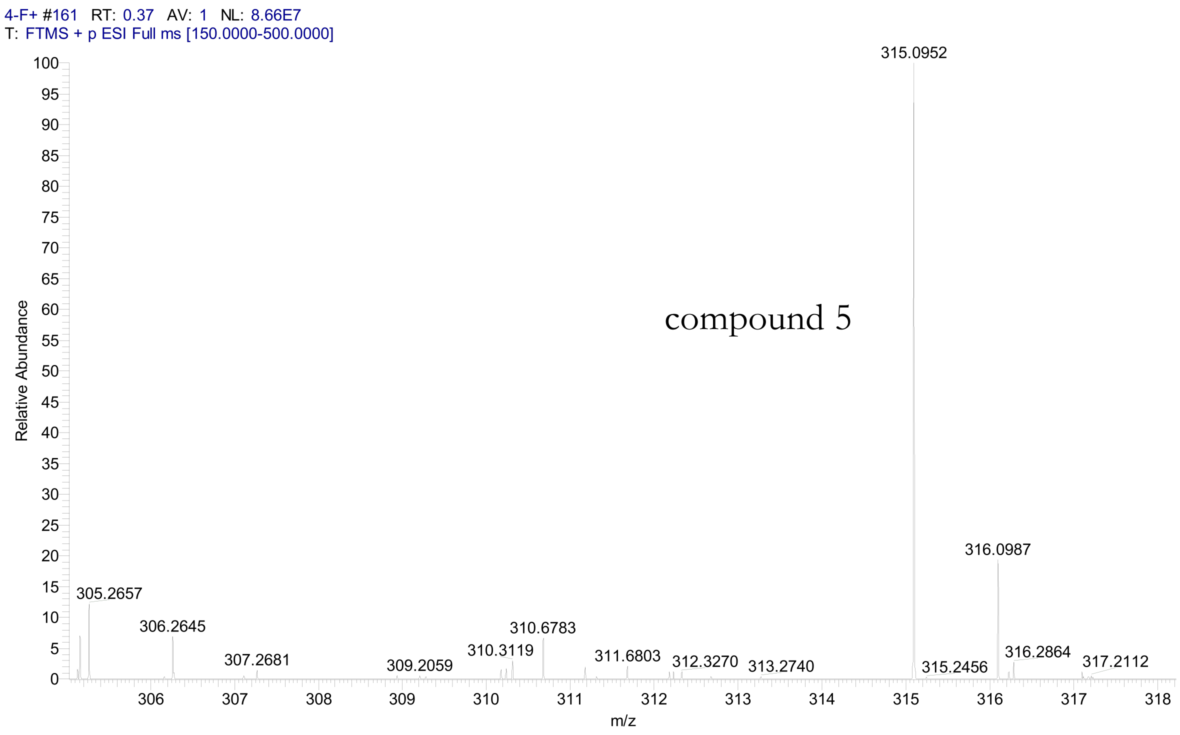
4-F+ #161 RT: 0.37 AV: 1 NL: 8.66E7
T: FTMS + p ESI Full ms [150.0000-500.0000]
Relative Abundance
m/z
315.0952
316.0987
305.2657
306.2645
307.2681
309.2059
310.3119
310.6783
311.6803
312.3270
313.2740
315.2456
316.2864
317.2112
compound 5

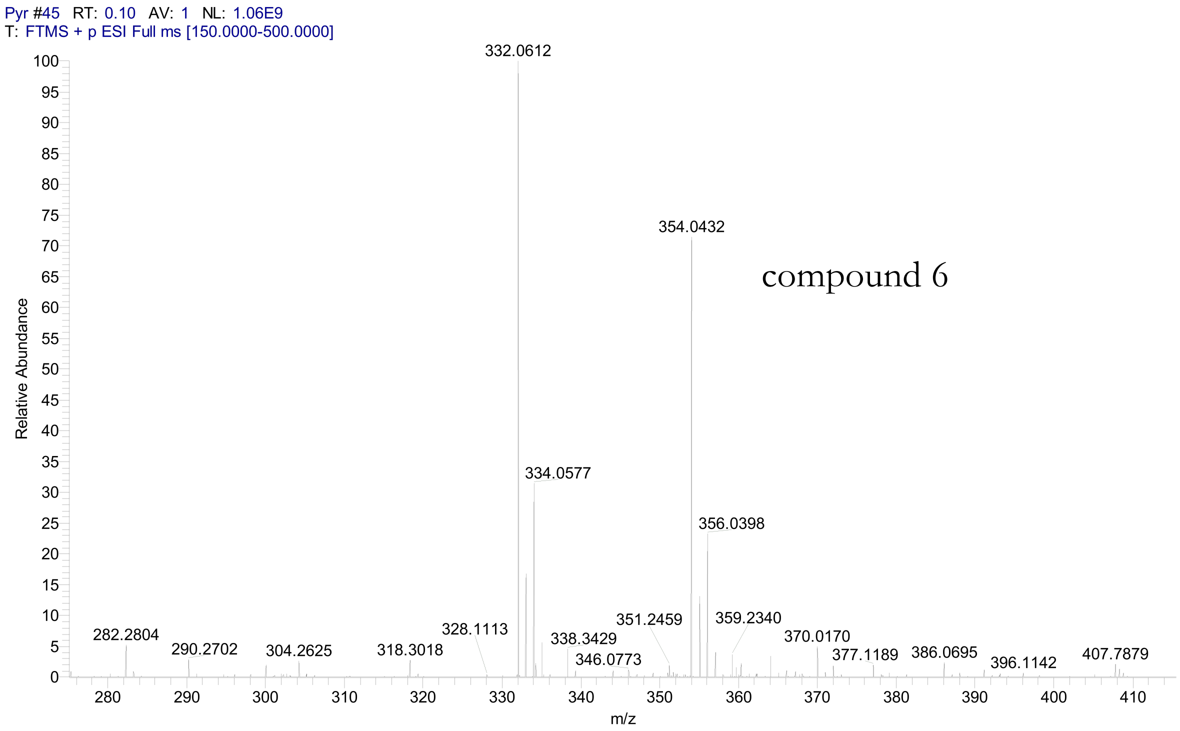

Pyr #45 RT: 0.10 AV: 1 NL: 1.06E9
T: FTMS + p ESI Full ms [150.0000-500.0000]
Relative Abundance
m/z
332.0612
354.0432
334.0577
356.0398
282.2804
290.2702
304.2625
318.3018
328.1113
338.3429
346.0773
351.2459
359.2340
370.0170
377.1189
386.0695
396.1142
407.7879

compound 6

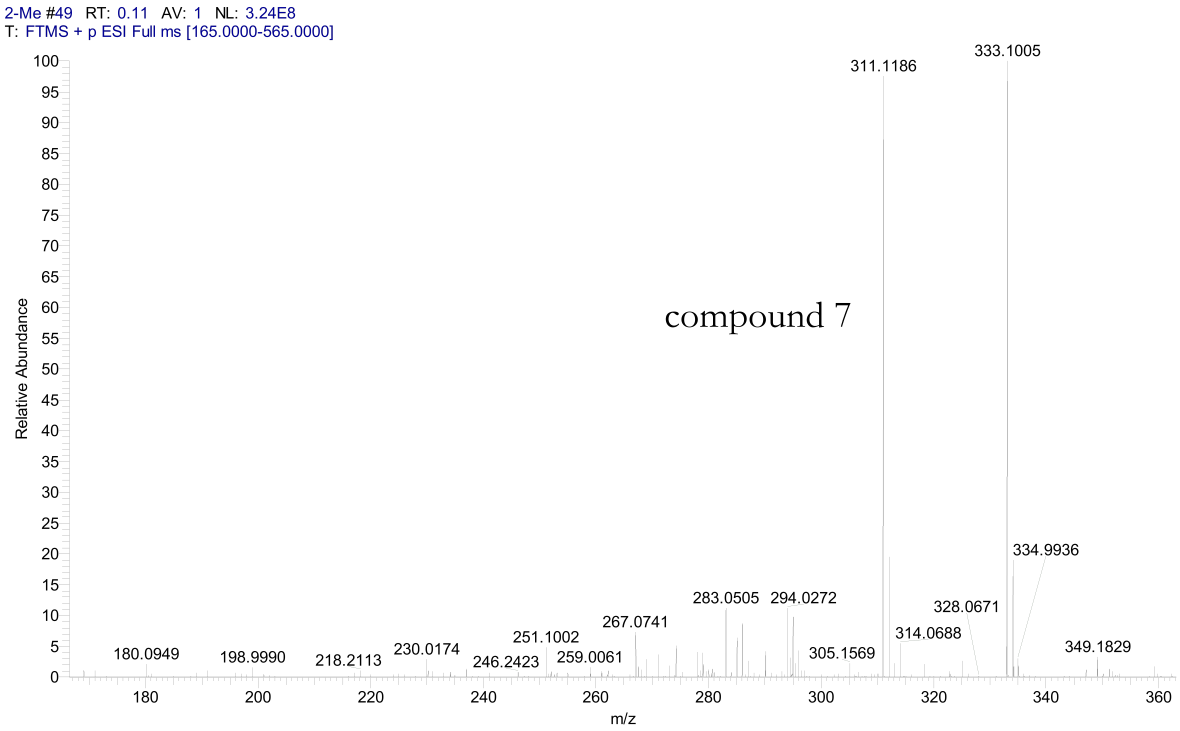
2-Me #49 RT: 0.11 AV: 1 NL: 3.24E8
T: FTMS + p ESI Full ms [165.0000-565.0000]
Relative Abundance
m/z
compound 7
311.1186
333.1005
334.9936
283.0505
294.0272
328.0671
267.0741
314.0688
251.1002
349.1829
180.0949
198.9990
218.2113
230.0174
246.2423
259.0061
305.1569

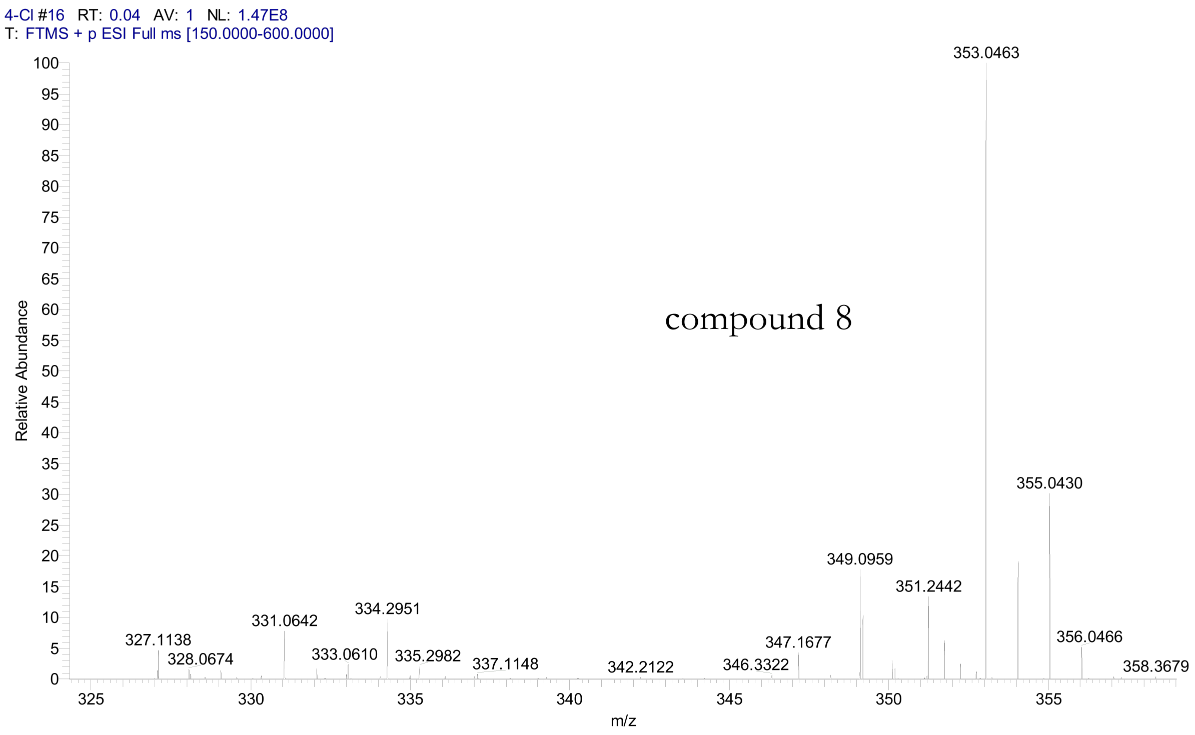

4-Cl #16 RT: 0.04 AV: 1 NL: 1.47E8
T: FTMS + p ESI Full ms [150.0000-600.0000]
compound 8
353.0463
355.0430
349.0959
351.2442
334.2951
331.0642
327.1138
328.0674
333.0610
335.2982
337.1148
342.2122
346.3322
347.1677
356.0466
358.3679
Relative Abundance
m/z

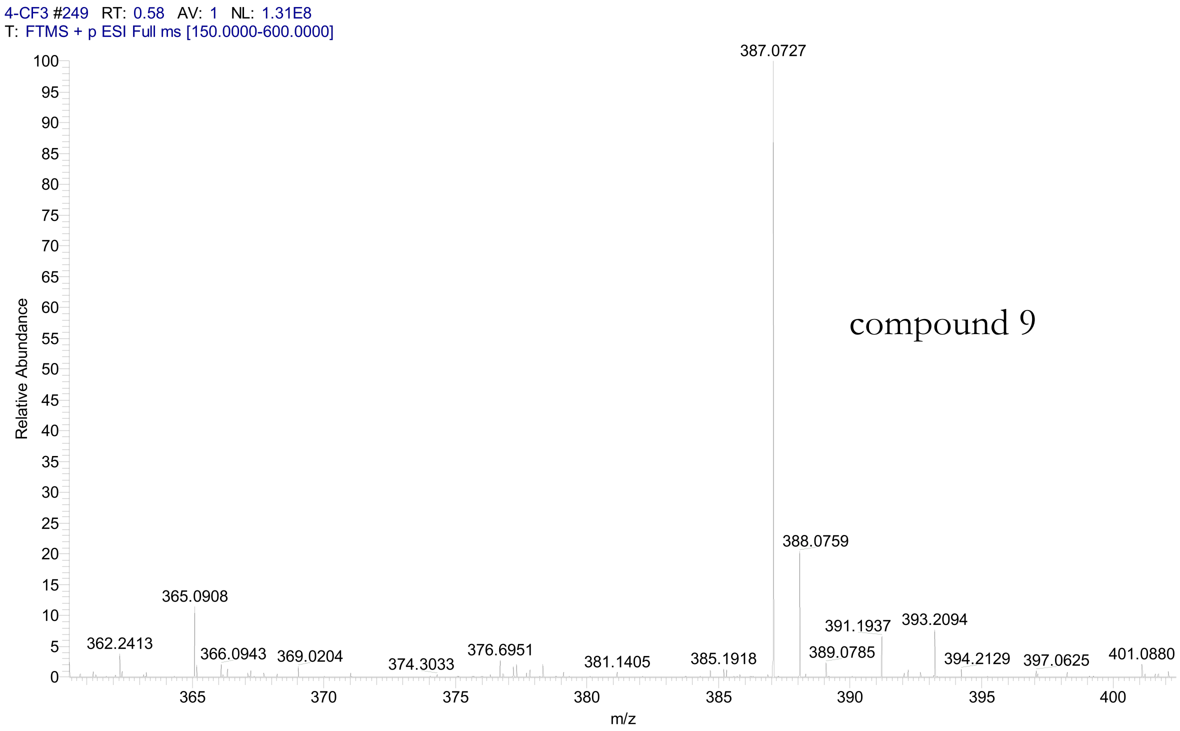
4-CF3 #249 RT: 0.58 AV: 1 NL: 1.31E8
T: FTMS + p ESI Full ms [150.0000-600.0000]
Relative Abundance
m/z
387.0727
388.0759
365.0908
362.2413
366.0943
369.0204
374.3033
376.6951
381.1405
385.1918
389.0785
391.1937
393.2094
394.2129
397.0625
401.0880

compound 9

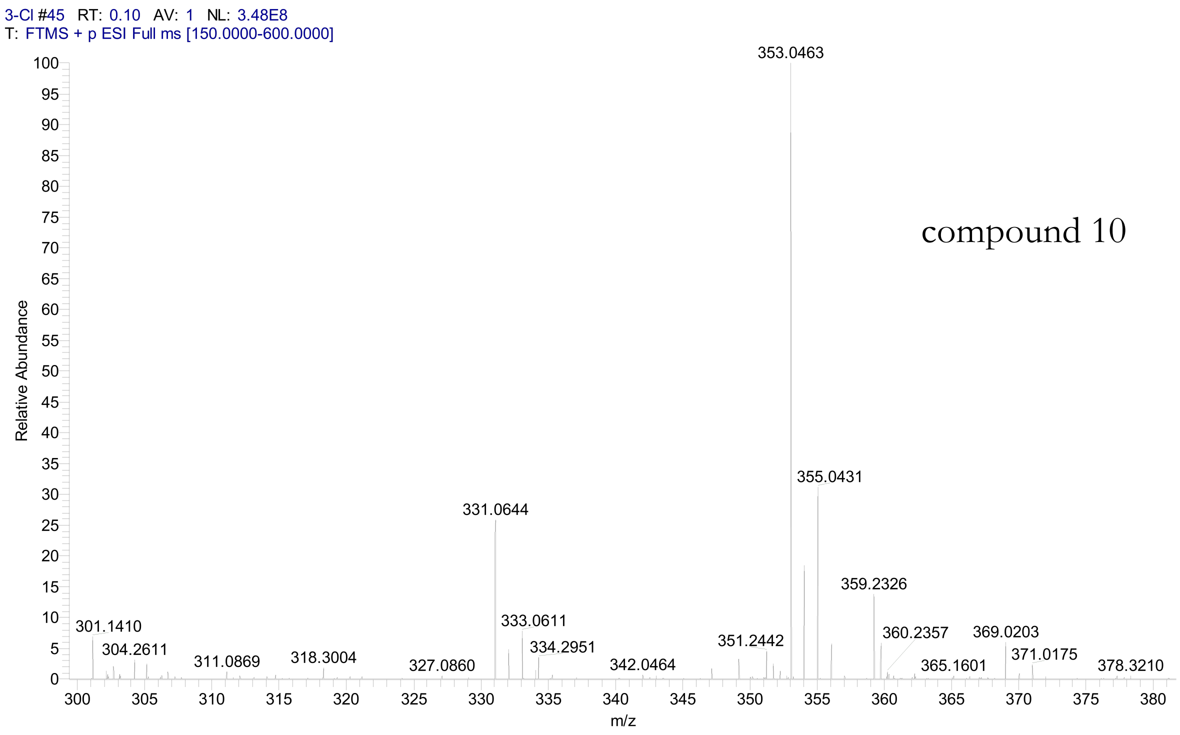
3-Cl #45 RT: 0.10 AV: 1 NL: 3.48E8
T: FTMS + p ESI Full ms [150.0000-600.0000]
compound 10
Relative Abundance
m/z
353.0463
355.0431
331.0644
359.2326
333.0611
301.1410
360.2357
369.0203
334.2951
351.2442
304.2611
371.0175
311.0869
318.3004
327.0860
342.0464
365.1601
378.3210

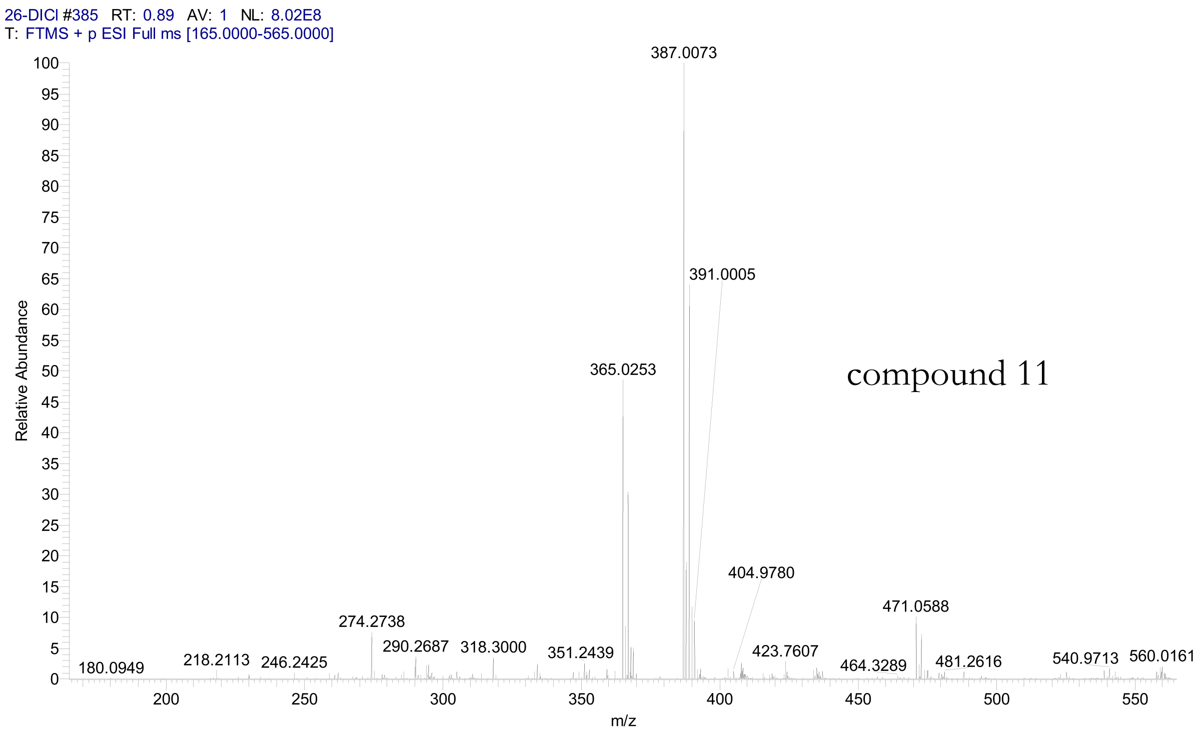
26-DICl #385 RT: 0.89 AV: 1 NL: 8.02E8
T: FTMS + p ESI Full ms [165.0000-565.0000]
387.0073
391.0005
365.0253
404.9780
471.0588
274.2738
290.2687
318.3000
423.7607
351.2439
180.0949
218.2113
246.2425
464.3289
481.2616
540.9713
560.0161
Relative Abundance
m/z
compound 11

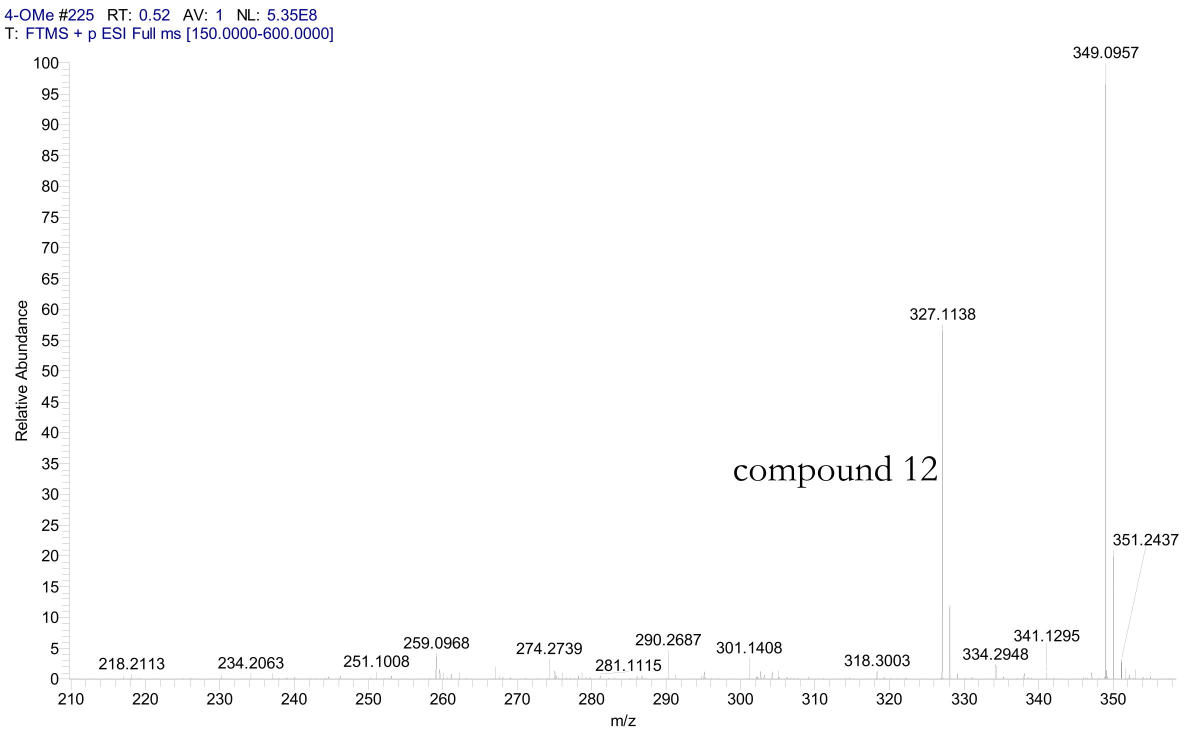
4-OMe #225 RT: 0.52 AV: 1 NL: 5.35E8
T: FTMS + p ESI Full ms [150.0000-600.0000]
Relative Abundance
m/z
349.0957
327.1138
351.2437
341.1295
334.2948
318.3003
301.1408
290.2687
281.1115
274.2739
259.0968
251.1008
234.2063
218.2113
compound 12

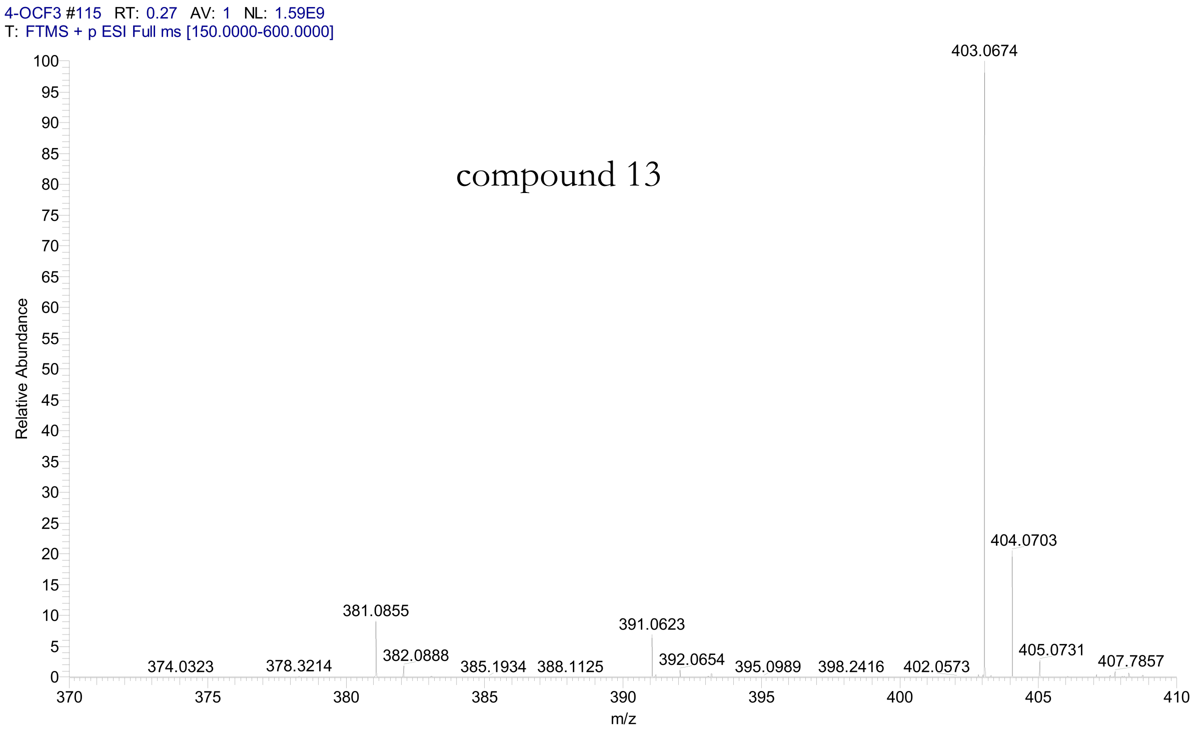
4-OCF3 #115 RT: 0.27 AV: 1 NL: 1.59E9
T: FTMS + p ESI Full ms [150.0000-600.0000]
compound 13
403.0674
404.0703
381.0855
391.0623
382.0888
392.0654
405.0731
407.7857
374.0323
378.3214
385.1934
388.1125
395.0989
398.2416
402.0573
Relative Abundance
m/z

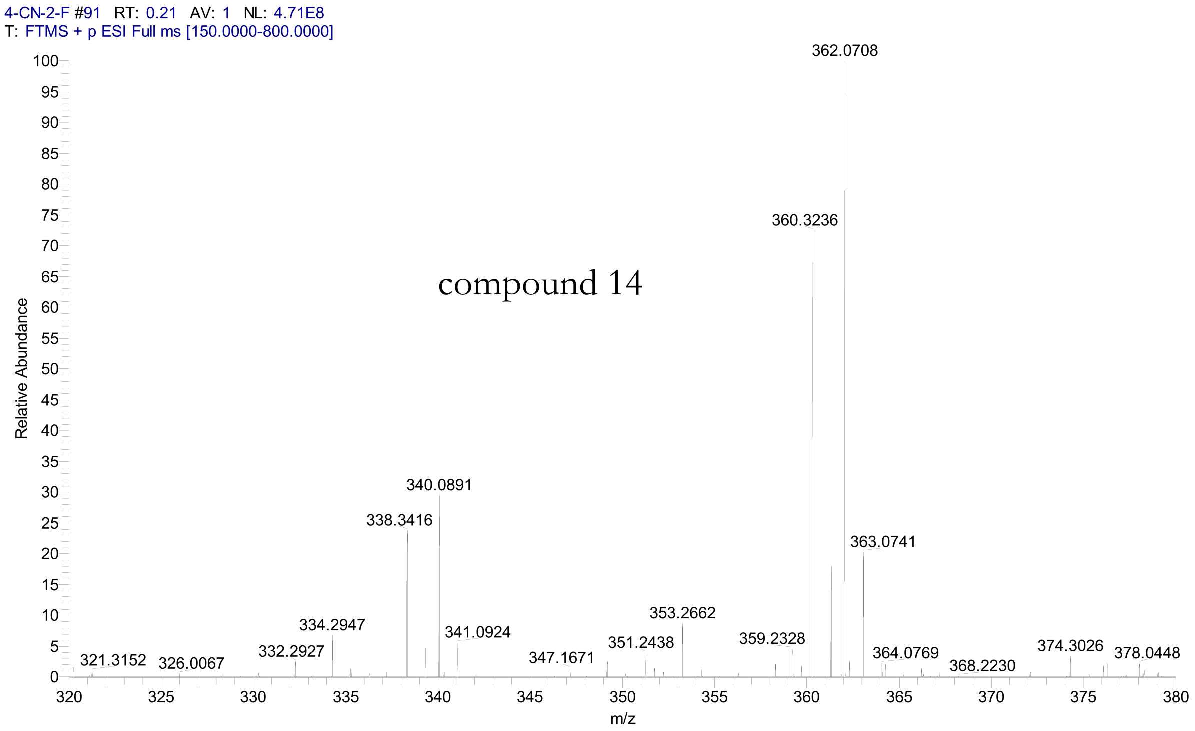
4-CN-2-F #91 RT: 0.21 AV: 1 NL: 4.71E8
T: FTMS + p ESI Full ms [150.0000-800.0000]
362.0708
360.3236
340.0891
338.3416
363.0741
353.2662
334.2947
341.0924
359.2328
351.2438
347.1671
321.3152
326.0067
332.2927
364.0769
368.2230
374.3026
378.0448
Relative Abundance
m/z

compound 14

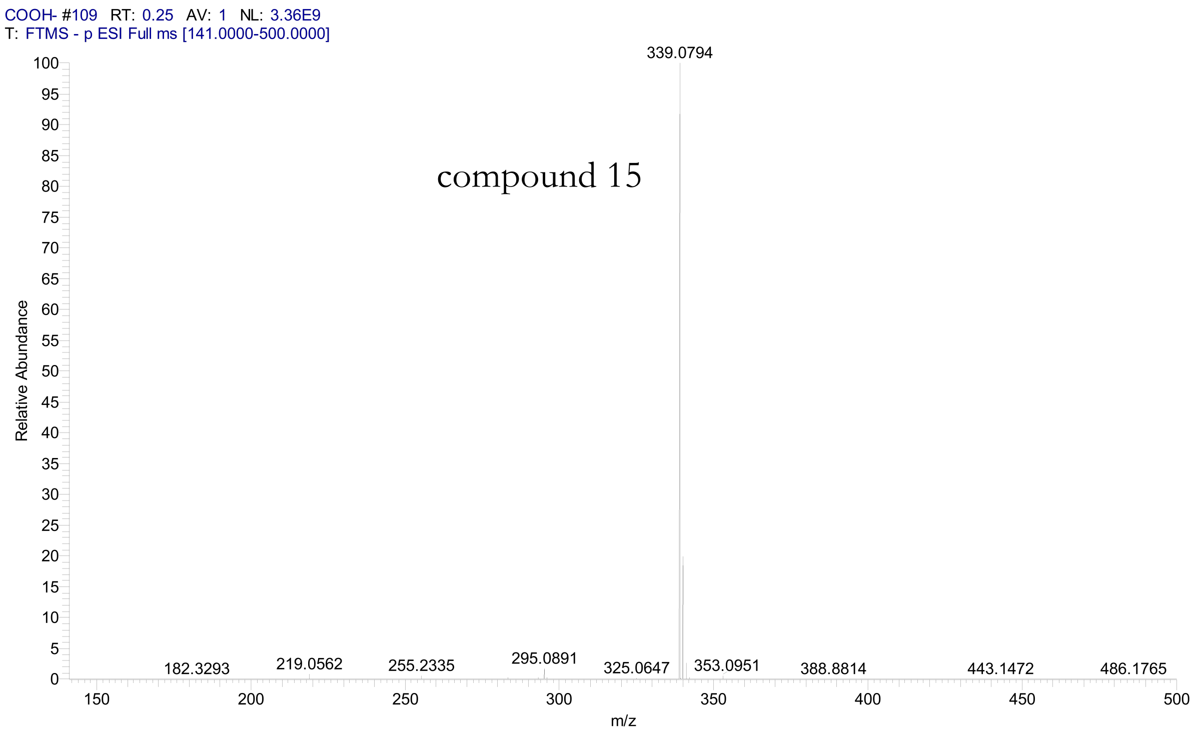
COOH- #109 RT: 0.25 AV: 1 NL: 3.36E9
T: FTMS - p ESI Full ms [141.0000-500.0000]
compound 15
339.0794
182.3293
219.0562
255.2335
295.0891
325.0647
353.0951
388.8814
443.1472
486.1765
Relative Abundance
m/z
