# Supplementary material for: Development of: 1,5-Diaryl-Pyrazole-3-Formate Analogs as Antifungal Pesticides and Their Application in Controlling Peanut Stem Rot Disease
Source: Front Microbiol. 2022 Jan 4;12:728173. doi: 10.3389/fmicb.2021.728173 (PMC8763808; doi:10.3389/fmicb.2021.728173)
Supplement: Supplementary file 2 [file Data_Sheet_2.PDF]

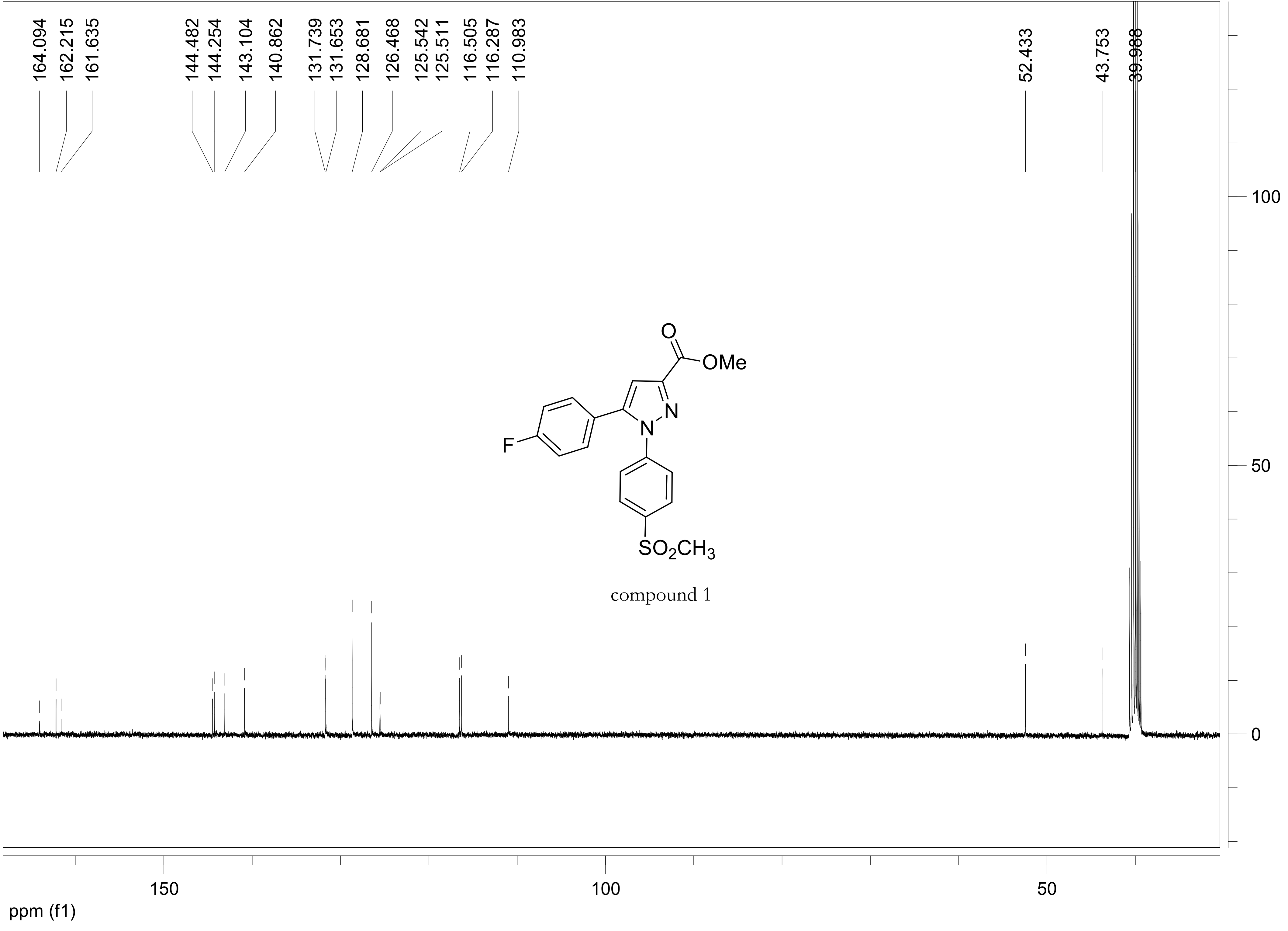

164.094
162.215
161.635
144.482
144.254
143.104
140.862
131.739
131.653
128.681
126.468
125.542
125.511
116.505
116.287
110.983
52.433
43.753
39.988
O
OMe
N
N
F
SO2CH3
compound 1
100
50
0
150
100
50
ppm (f1)

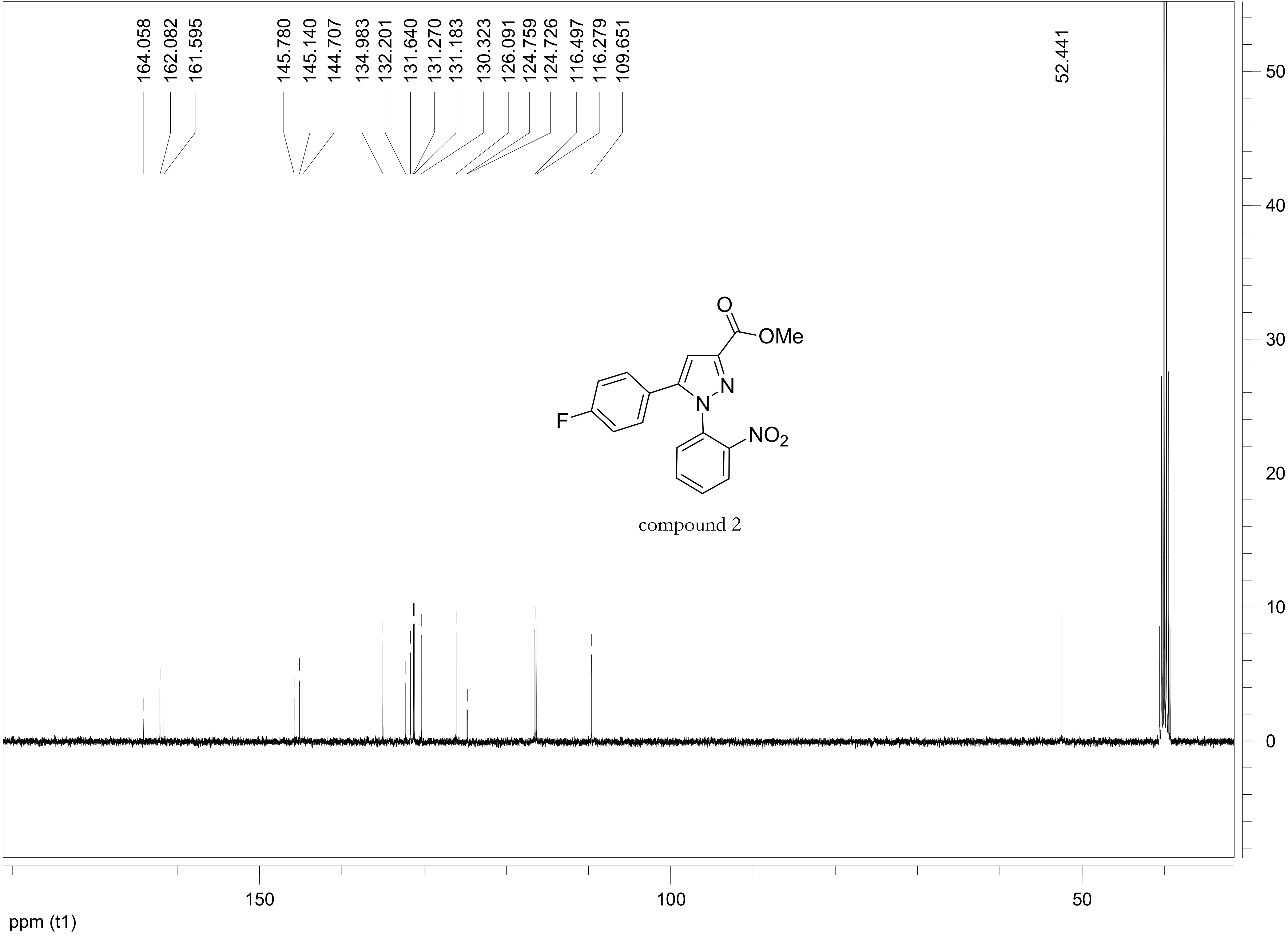
164.058
162.082
161.595
145.780
145.140
144.707
134.983
132.201
131.640
131.270
131.183
130.323
126.091
124.759
124.726
116.497
116.279
109.651
52.441
O
OMe
N
N
F
NO2
150
100
50
ppm (t1)
50
40
30
20
10
0

compound 2

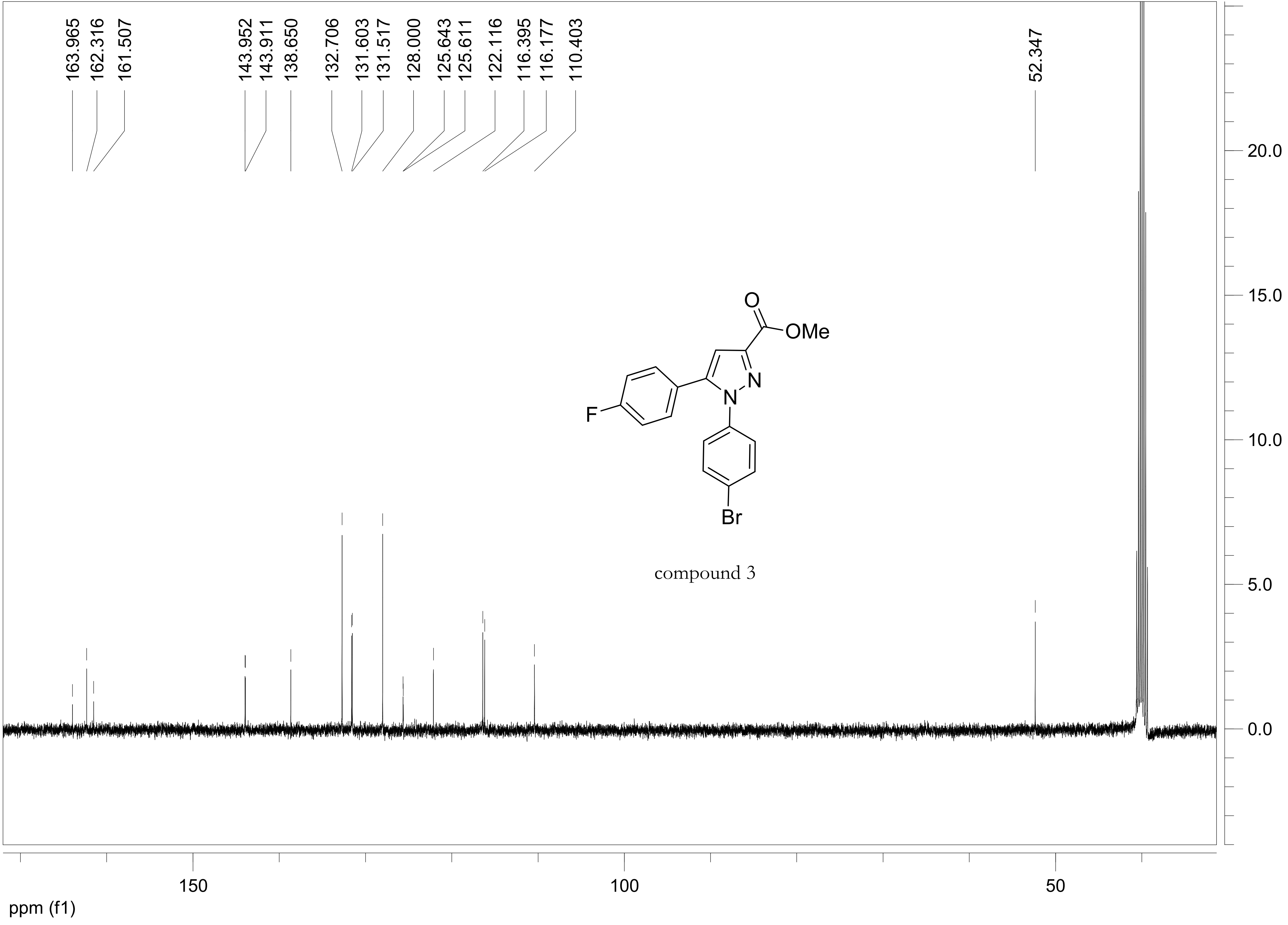
163.965
162.316
161.507
143.952
143.911
138.650
132.706
131.603
131.517
128.000
125.643
125.611
122.116
116.395
116.177
110.403
52.347
O
OMe
N
N
F
Br
compound 3
20.0
15.0
10.0
5.0
0.0
150
100
50
ppm (f1)

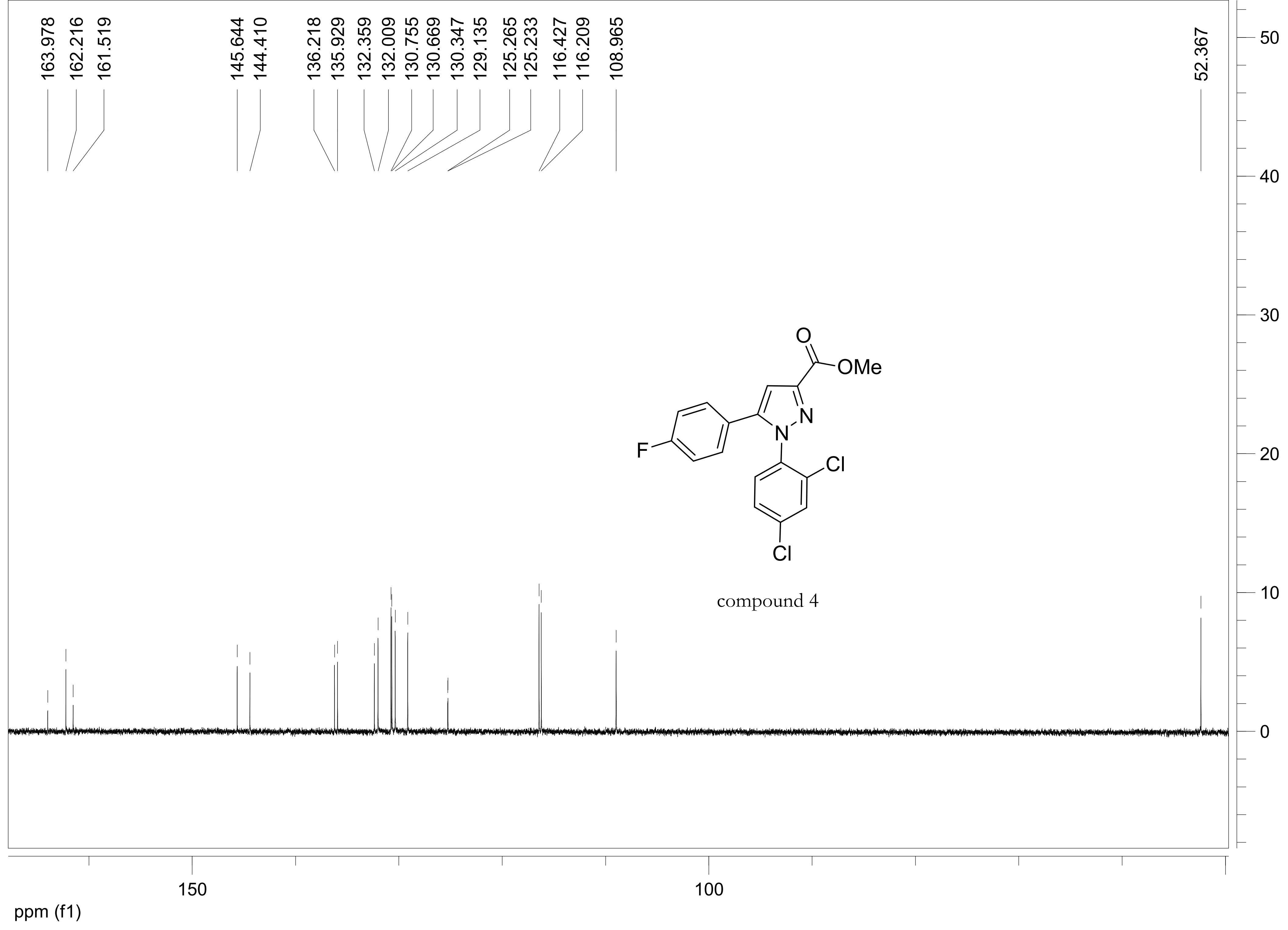

163.978
162.216
161.519
145.644
144.410
136.218
135.929
132.359
132.009
130.755
130.669
130.347
129.135
125.265
125.233
116.427
116.209
108.965
52.367
O
OMe
N
N
F
Cl
Cl
compound 4
50
40
30
20
10
0
150
100
ppm (f1)

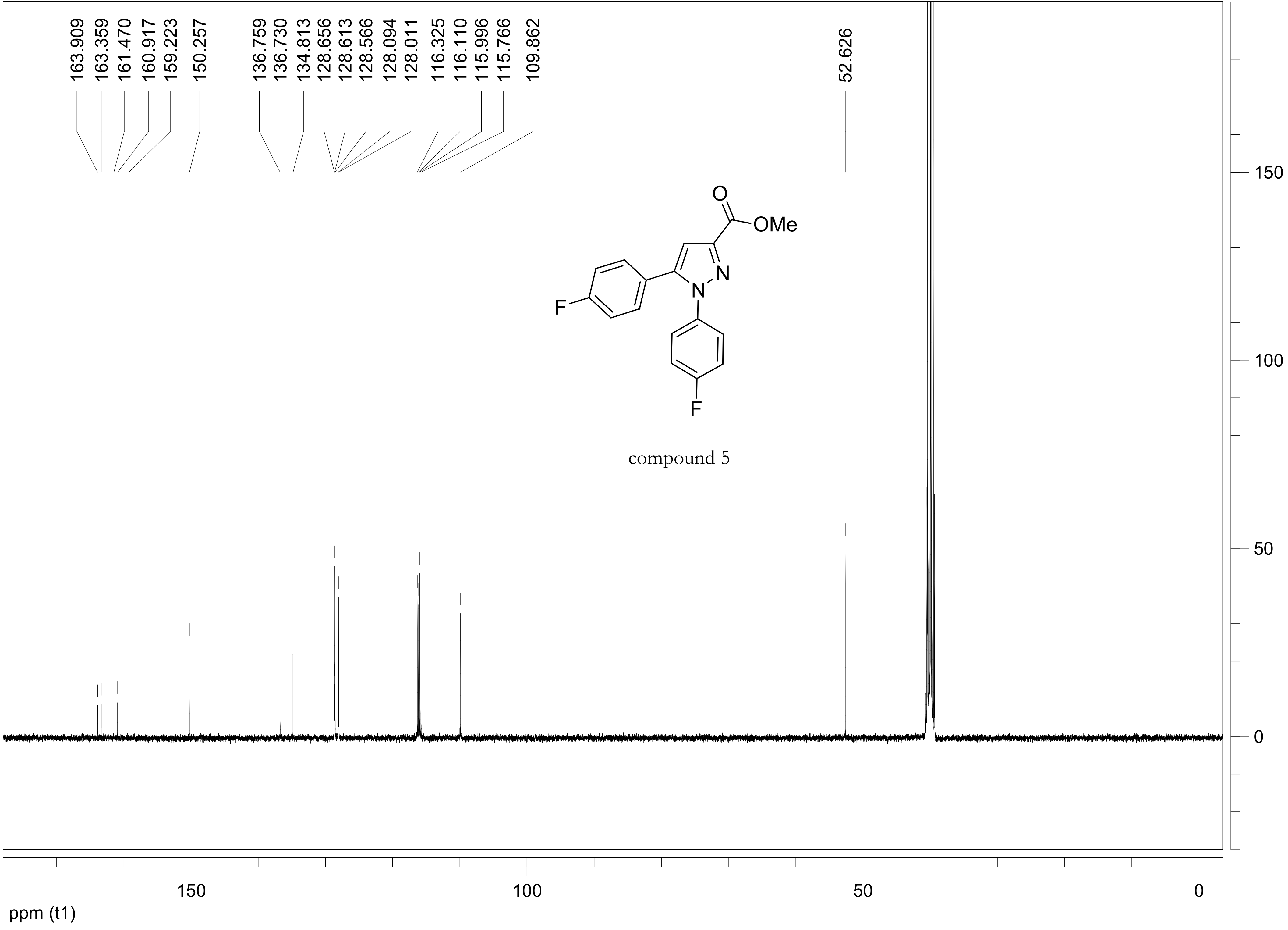
163.909
163.359
161.470
160.917
159.223
150.257
136.759
136.730
134.813
128.656
128.613
128.566
128.094
128.011
116.325
116.110
115.996
115.766
109.862
52.626
O
OMe
N
N
F
F
compound 5
150
100
50
0
ppm (t1)

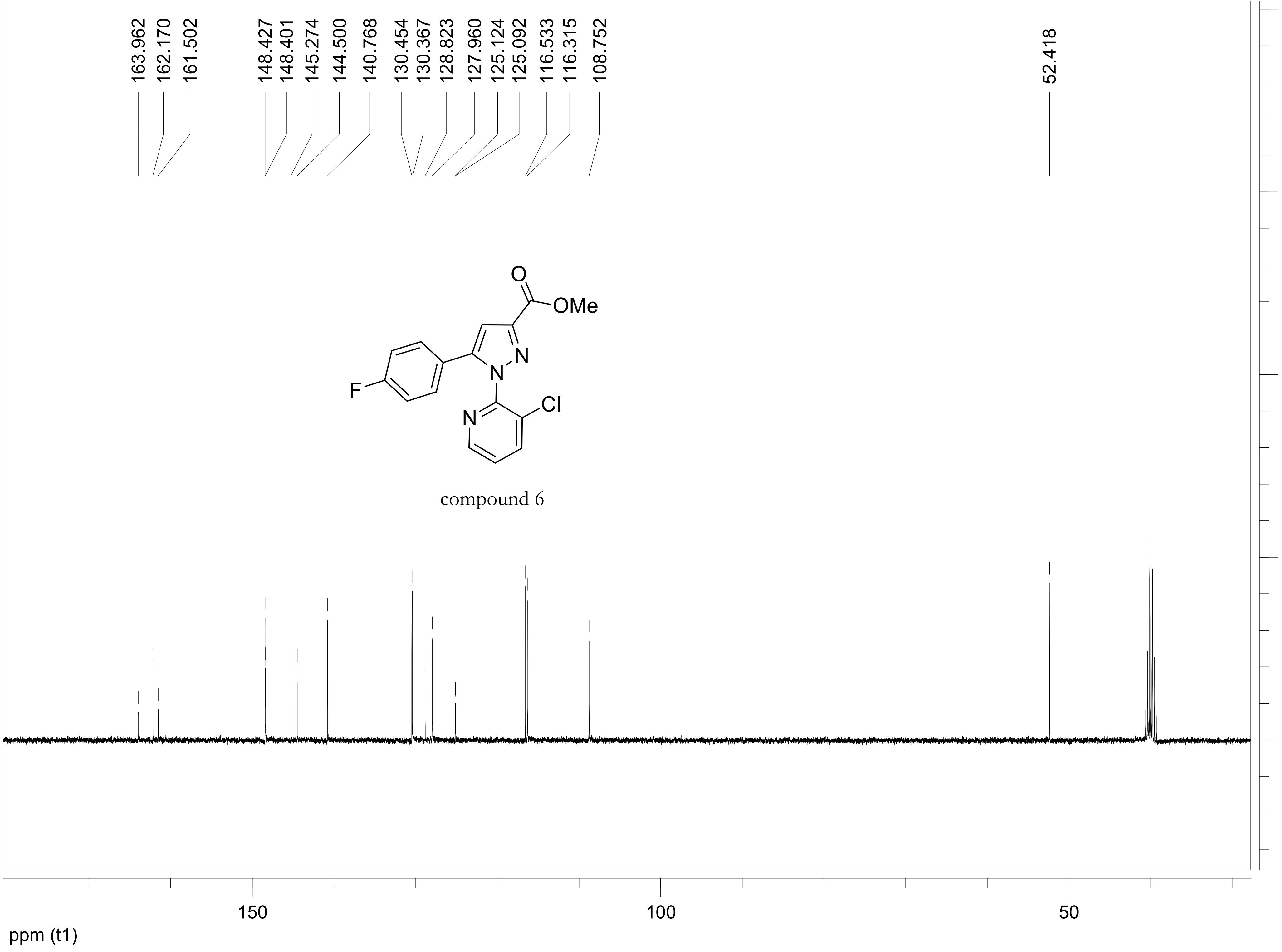
163.962
162.170
161.502
148.427
148.401
145.274
144.500
140.768
130.454
130.367
128.823
127.960
125.124
125.092
116.533
116.315
108.752
52.418
O
OMe
N
N
F
N
Cl
compound 6
30
20
10
0
150
100
50
ppm (t1)

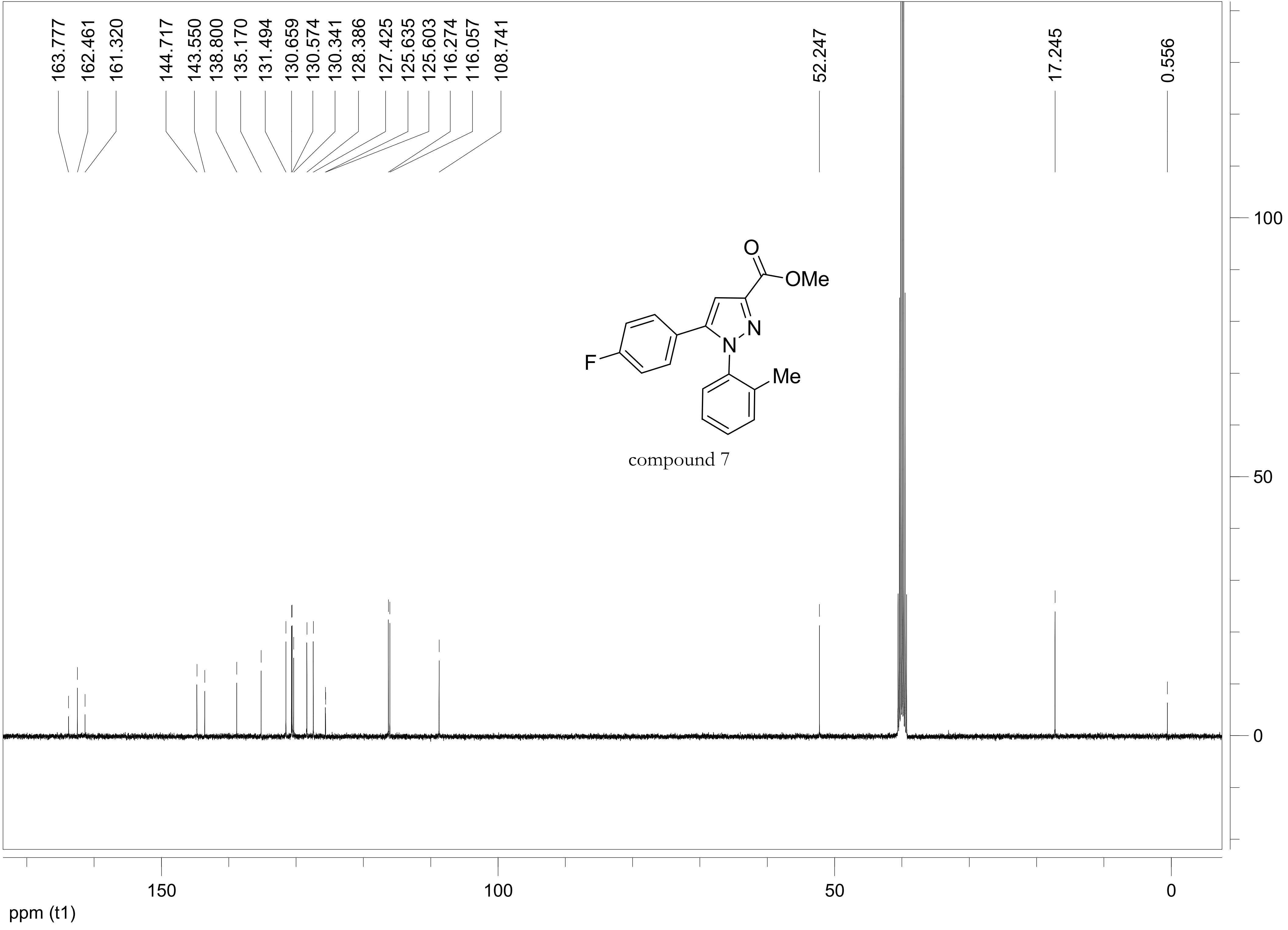

163.777
162.461
161.320
144.717
143.550
138.800
135.170
131.494
130.659
130.574
130.341
128.386
127.425
125.635
125.603
116.274
116.057
108.741
52.247
17.245
0.556
O
OMe
N
N
F
Me
compound 7
100
50
0
150
100
50
0
ppm (t1)

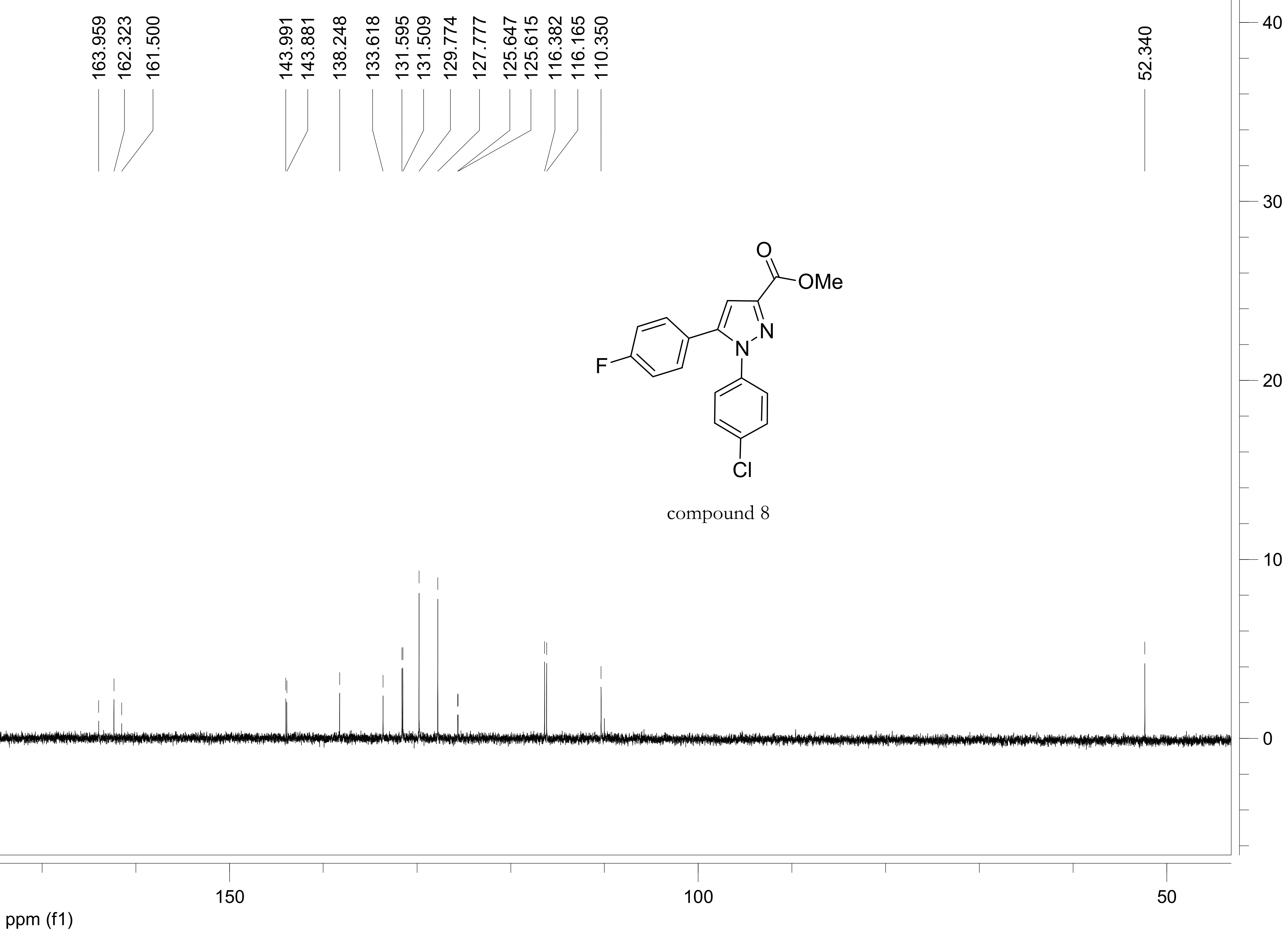
163.959
162.323
161.500
143.991
143.881
138.248
133.618
131.595
131.509
129.774
127.777
125.647
125.615
116.382
116.165
110.350
52.340
O
OMe
N
N
F
Cl
compound 8
40
30
20
10
0
150
100
50
ppm (f1)

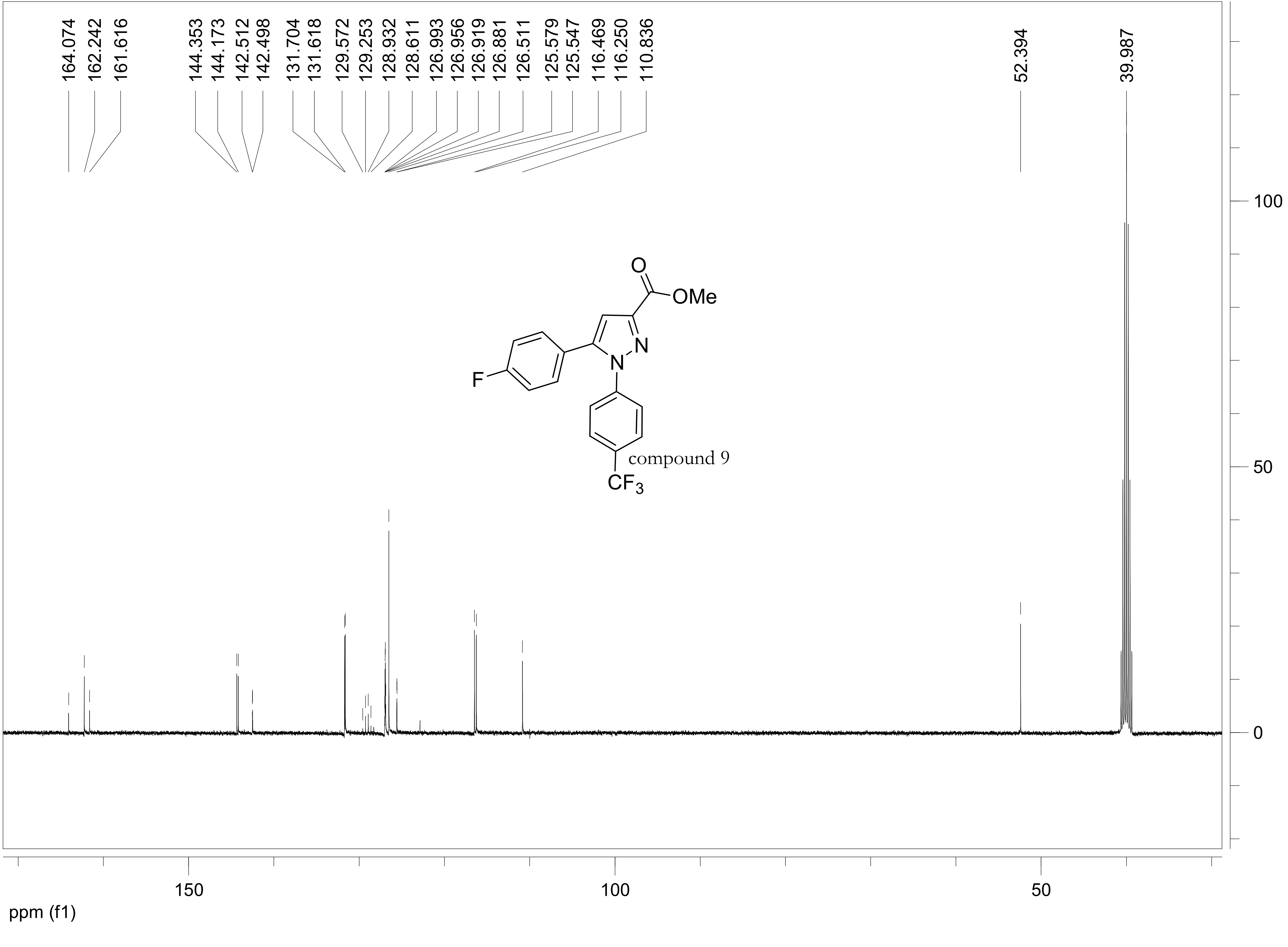
164.074
162.242
161.616
144.353
144.173
142.512
142.498
131.704
131.618
129.572
129.253
128.932
128.611
126.993
126.956
126.919
126.881
126.511
125.579
125.547
116.469
116.250
110.836
52.394
39.987
O
OMe
N
N
F
CF3
compound 9
100
50
0
150
100
50
ppm (f1)

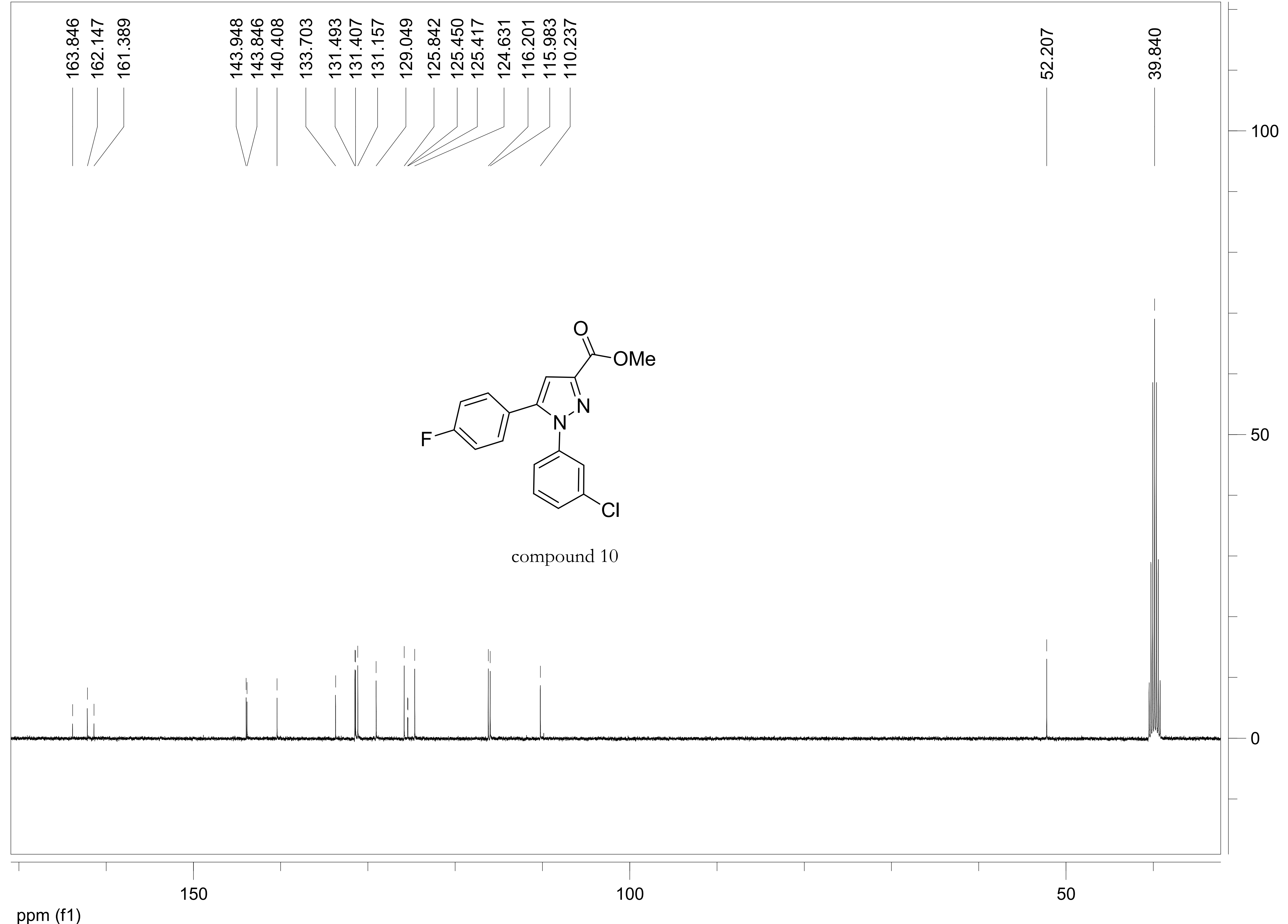
163.846
162.147
161.389
143.948
143.846
140.408
133.703
131.493
131.407
131.157
129.049
125.842
125.450
125.417
124.631
116.201
115.983
110.237
52.207
39.840
O
OMe
N
N
F
Cl
compound 10
100
50
0
150
100
50
ppm (f1)

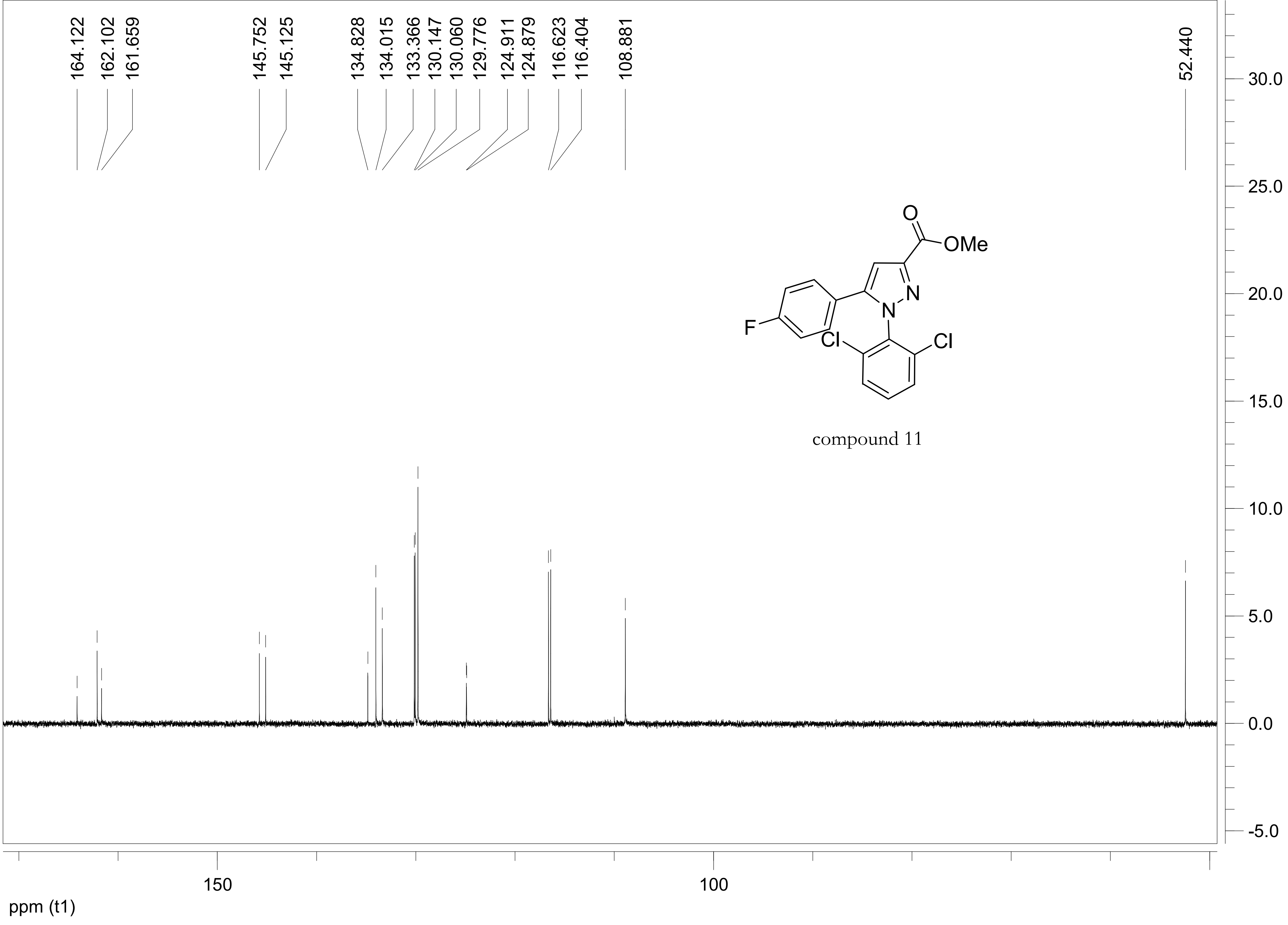
164.122
162.102
161.659
145.752
145.125
134.828
134.015
133.366
130.147
130.060
129.776
124.911
124.879
116.623
116.404
108.881
52.440
O
OMe
N
N
F
Cl
Cl
compound 11
30.0
25.0
20.0
15.0
10.0
5.0
0.0
-5.0
150
100
ppm (t1)

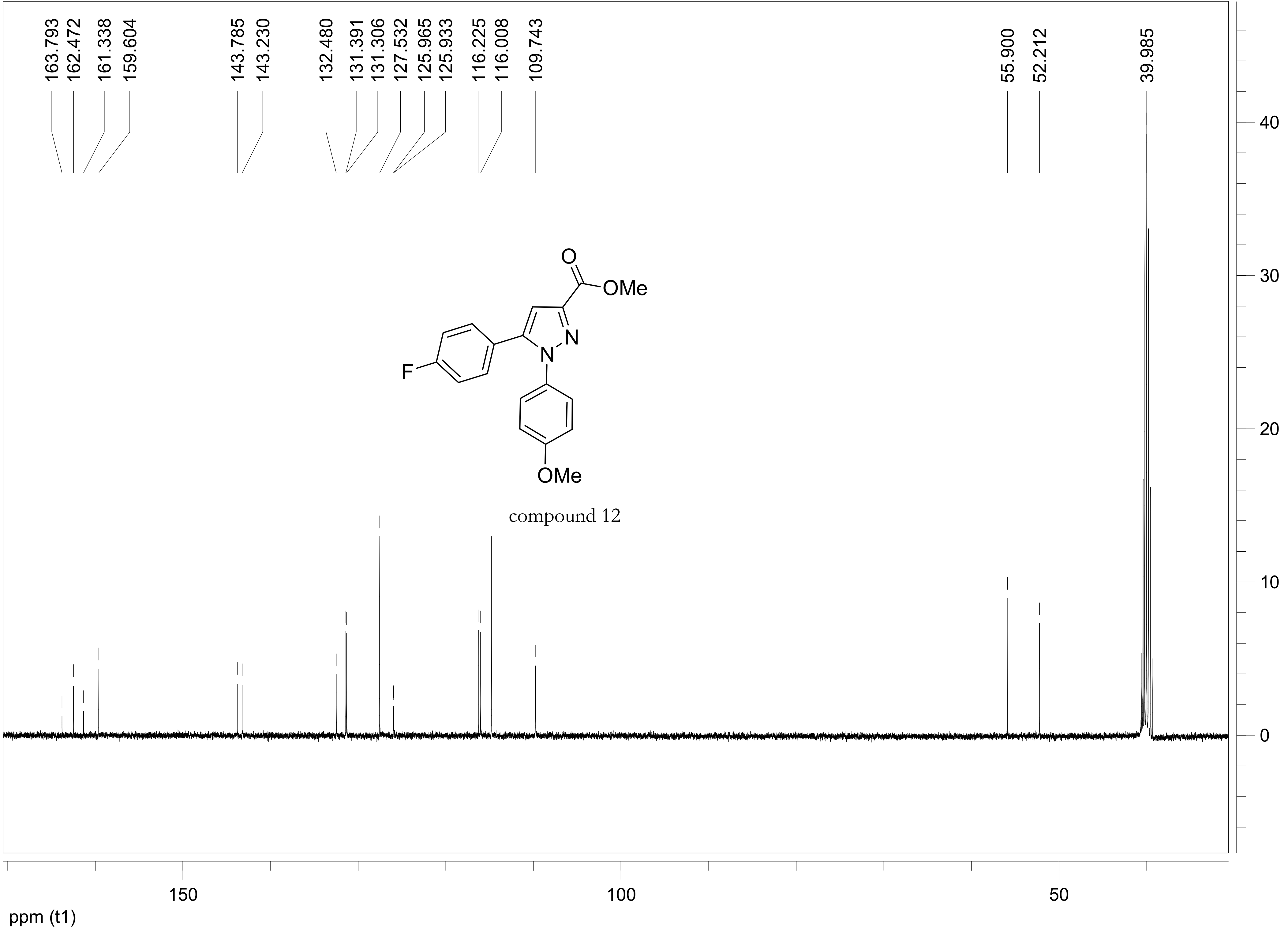
163.793
162.472
161.338
159.604
143.785
143.230
132.480
131.391
131.306
127.532
125.965
125.933
116.225
116.008
109.743
55.900
52.212
39.985
O
OMe
N
N
F
OMe
40
30
20
10
0
150
100
50
ppm (t1)
compound 12

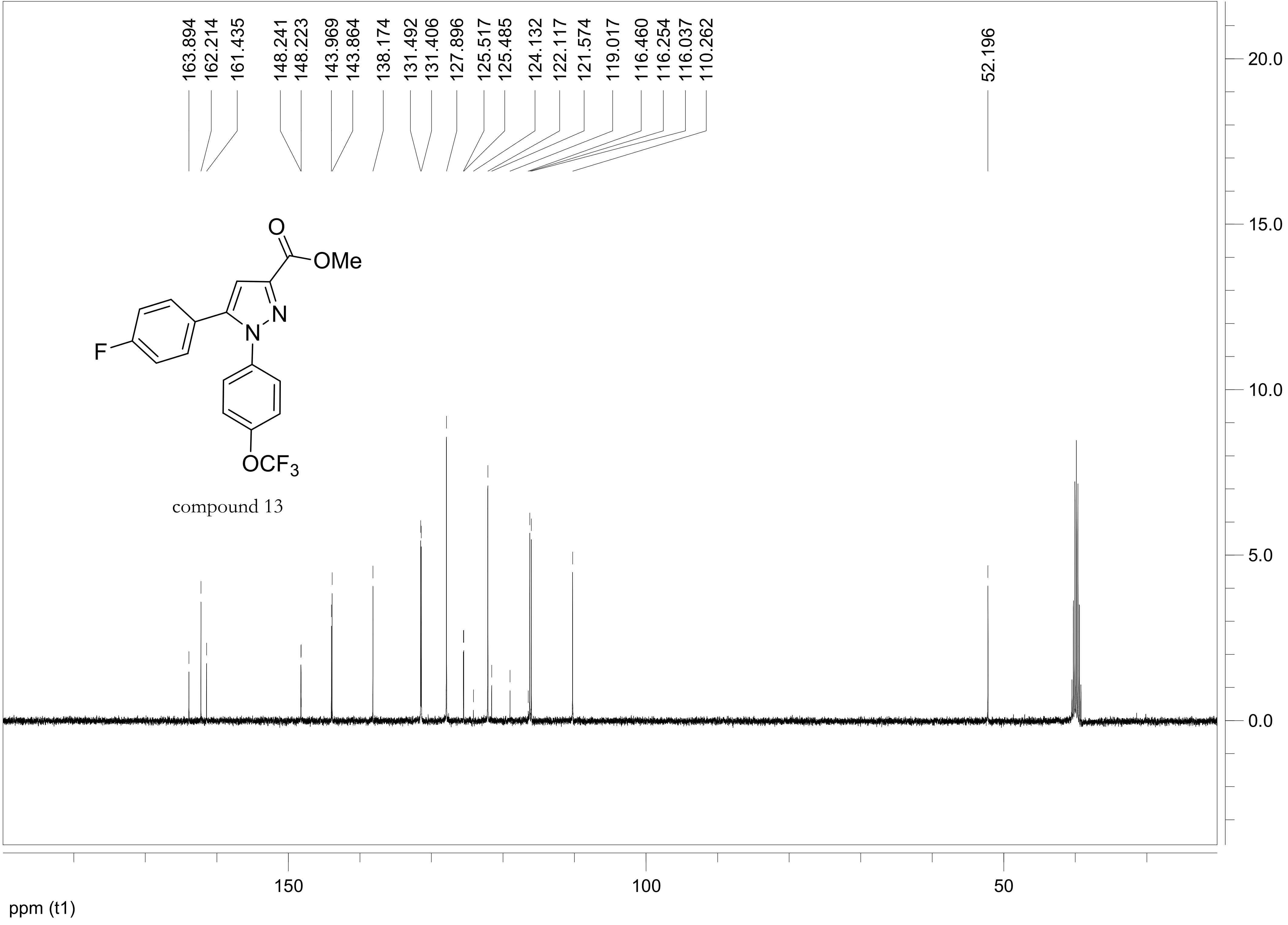

163.894
162.214
161.435
148.241
148.223
143.969
143.864
138.174
131.492
131.406
127.896
125.517
125.485
124.132
122.117
121.574
119.017
116.460
116.254
116.037
110.262
52.196
O
OMe
N
N
F
OCF3
20.0
15.0
10.0
5.0
0.0
150
100
50
ppm (t1)

compound 13

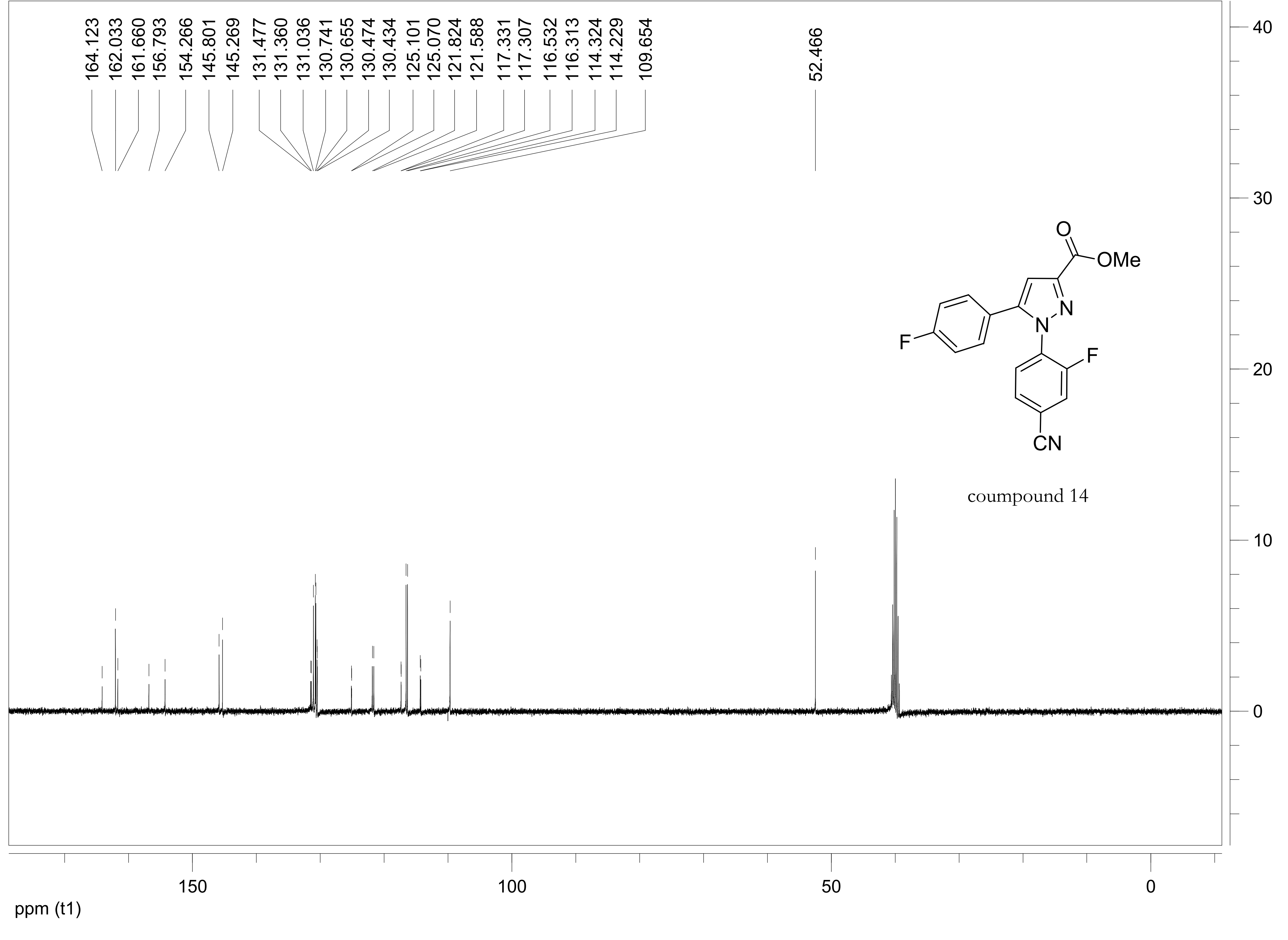
164.123
162.033
161.660
156.793
154.266
145.801
145.269
131.477
131.360
131.036
130.741
130.655
130.474
130.434
125.101
125.070
121.824
121.588
117.331
117.307
116.532
116.313
114.324
114.229
109.654
52.466
O
OMe
N
N
F
F
CN
coumpound 14
40
30
20
10
0
150
100
50
0
ppm (t1)

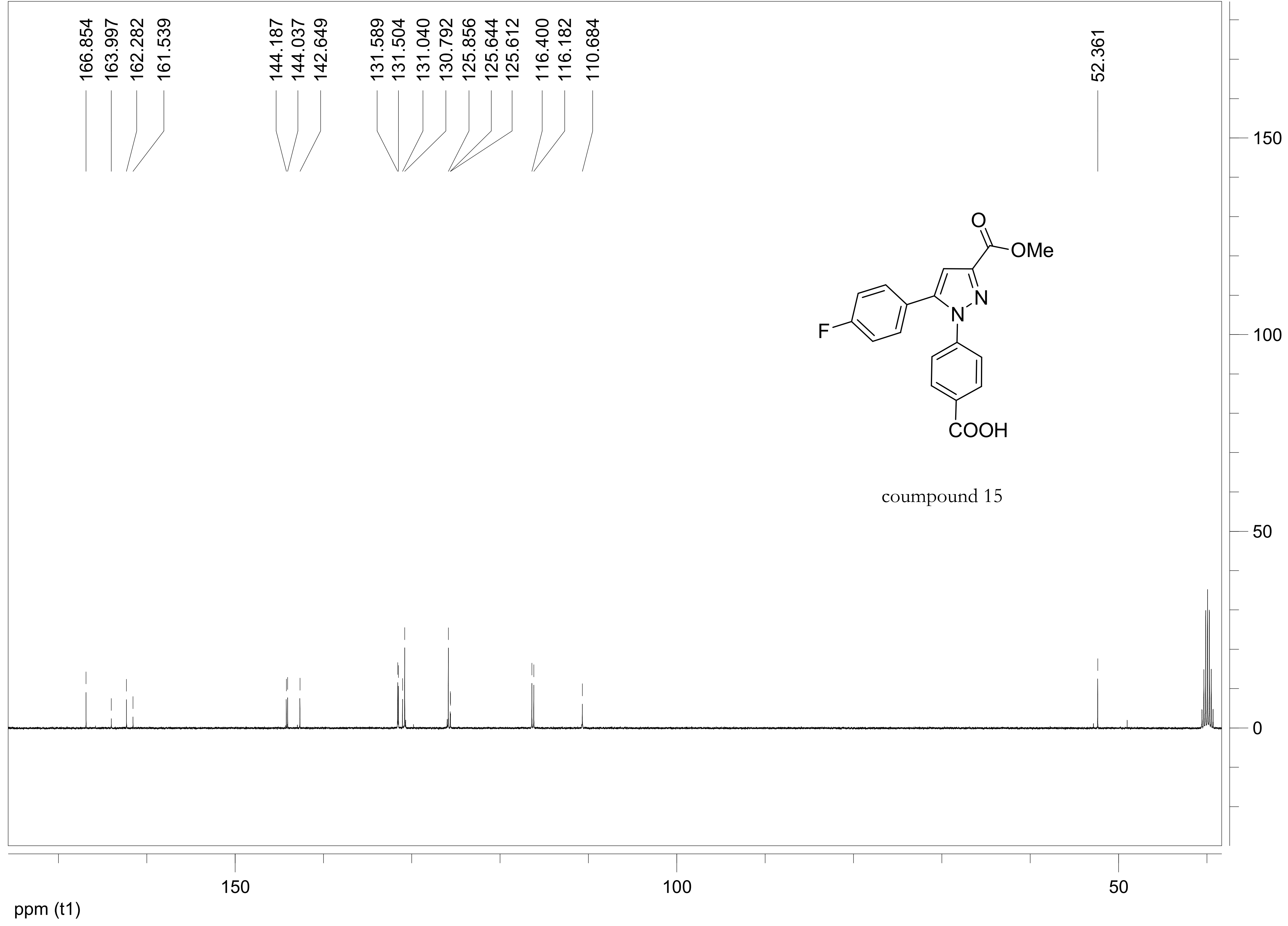

166.854
163.997
162.282
161.539
144.187
144.037
142.649
131.589
131.504
131.040
130.792
125.856
125.644
125.612
116.400
116.182
110.684
52.361
O
OMe
N
N
F
COOH
coumpound 15
150
100
50
0
ppm (t1)
