# Supplementary figures and images for: Development of: 1,5-Diaryl-Pyrazole-3-Formate Analogs as Antifungal Pesticides and Their Application in Controlling Peanut Stem Rot Disease
Source: Front Microbiol. 2022 Jan 4;12:728173. doi: 10.3389/fmicb.2021.728173 (PMC8763808; doi:10.3389/fmicb.2021.728173)

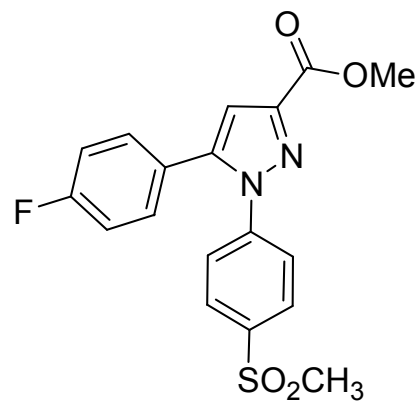

compound 1

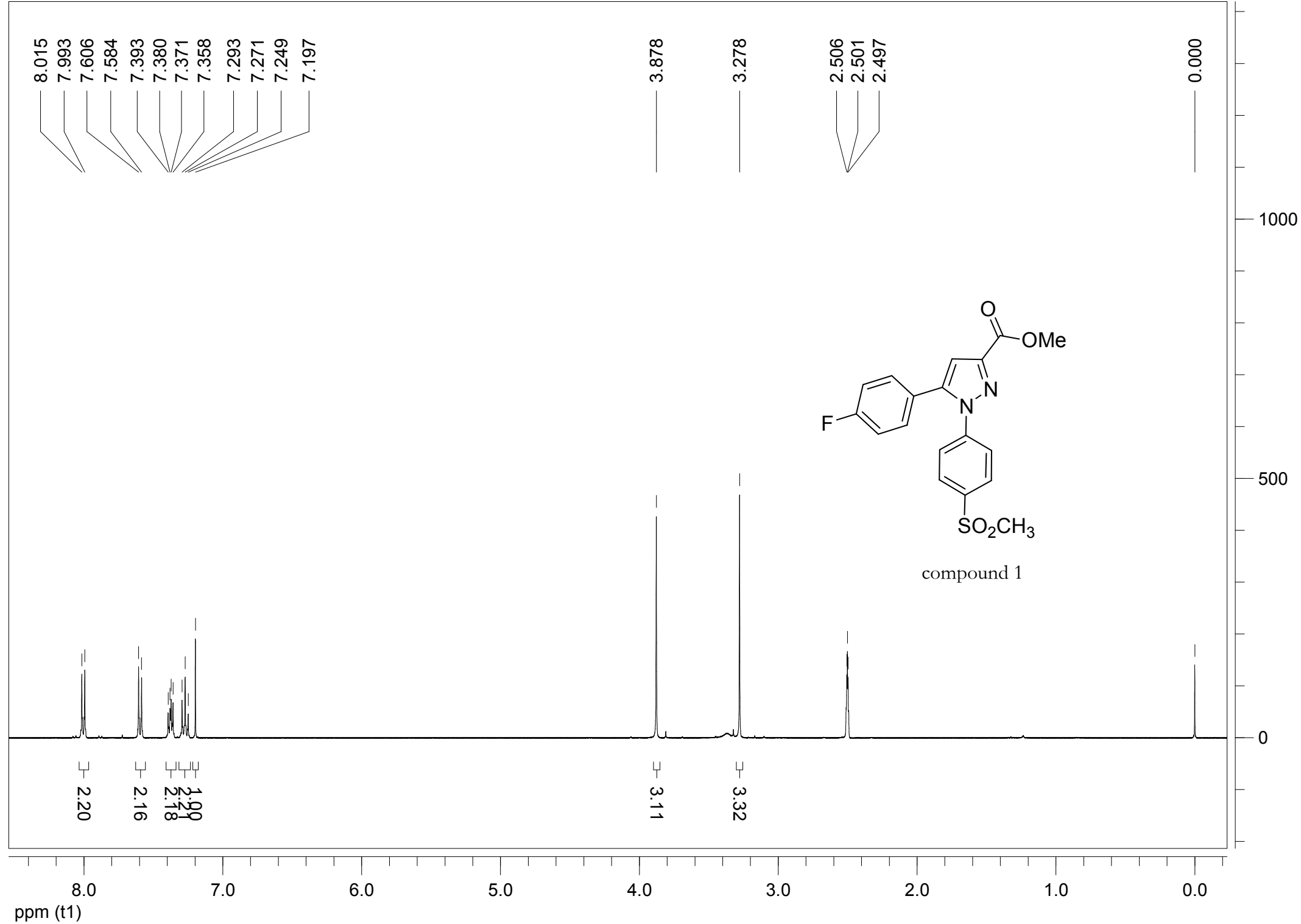

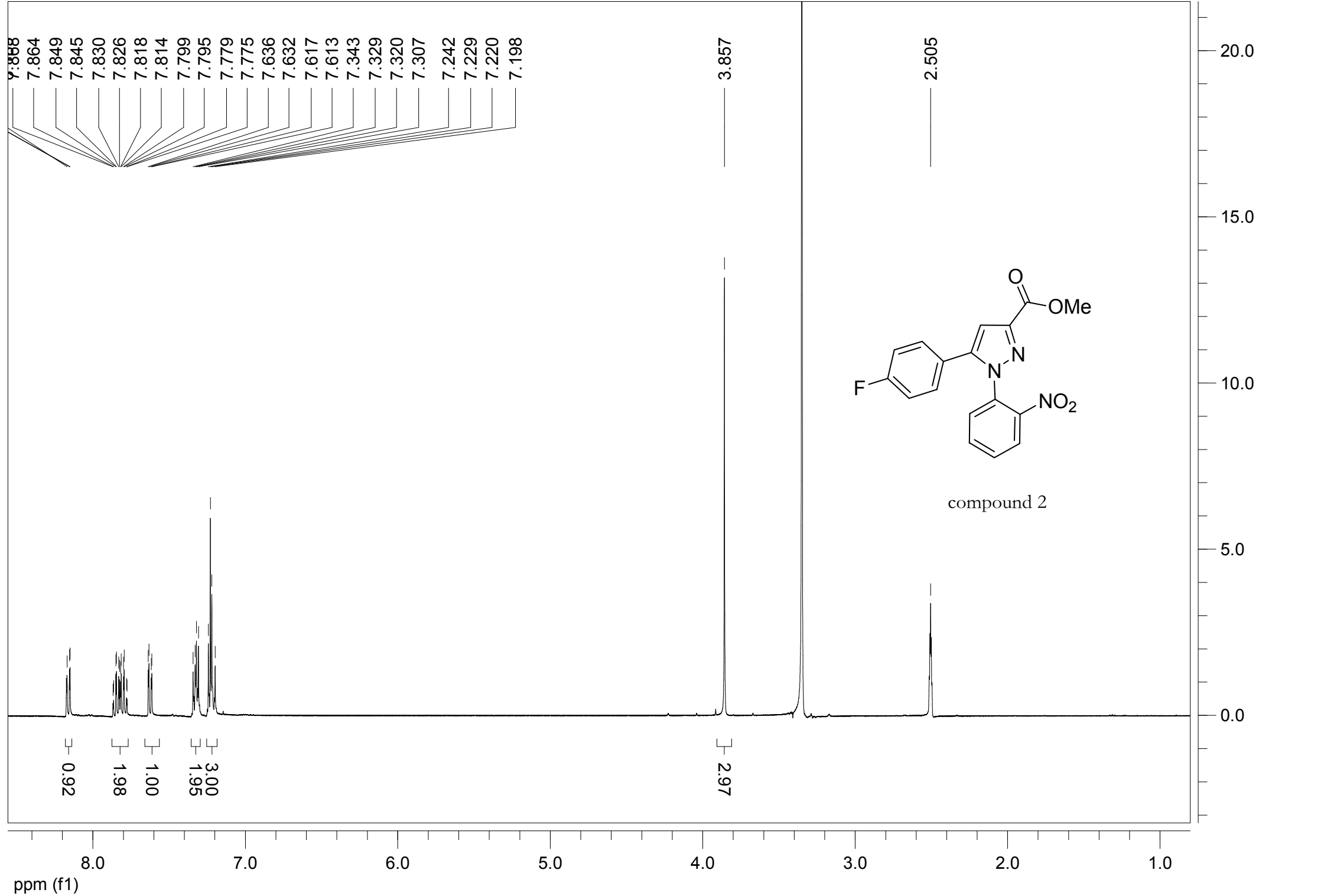

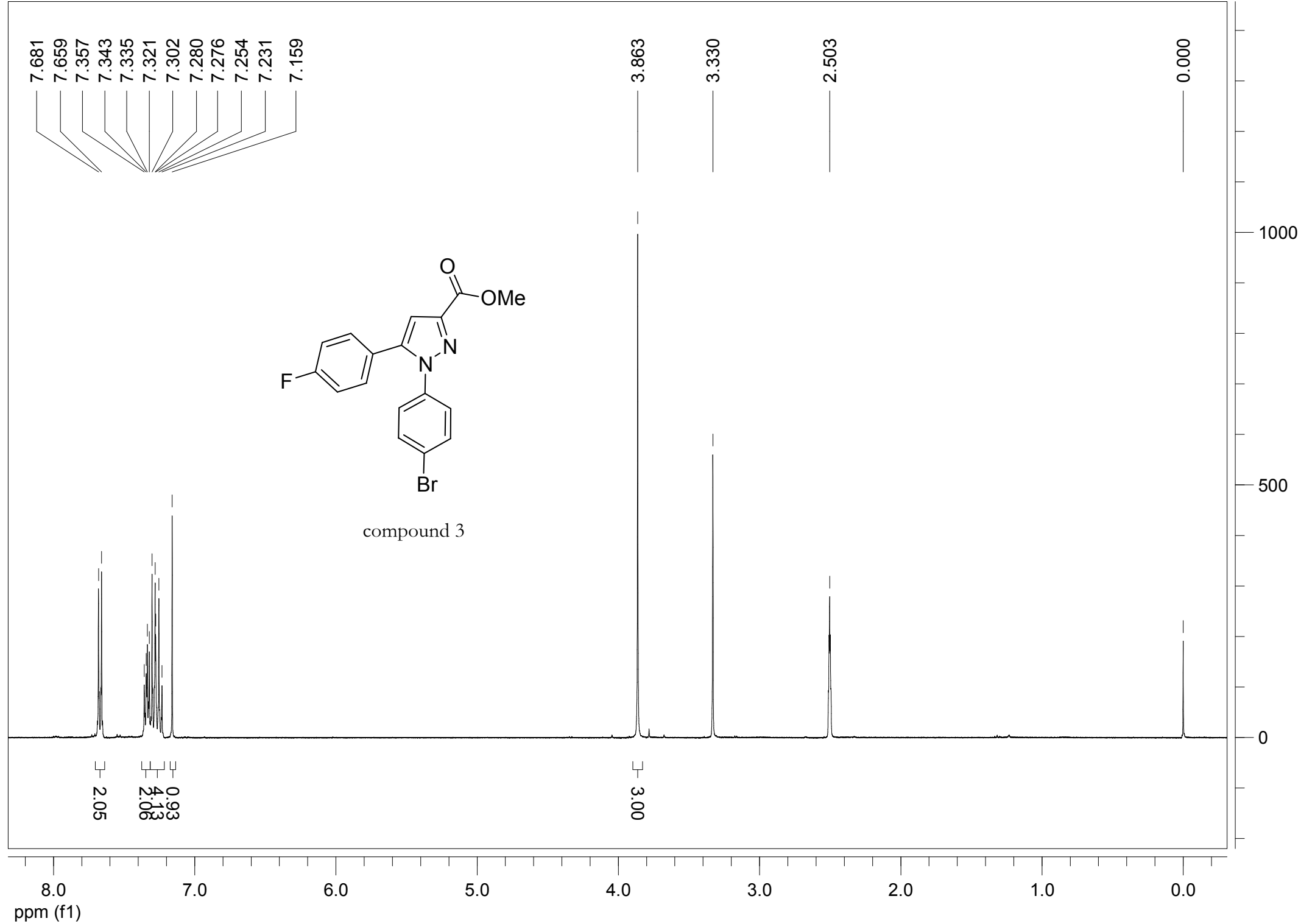

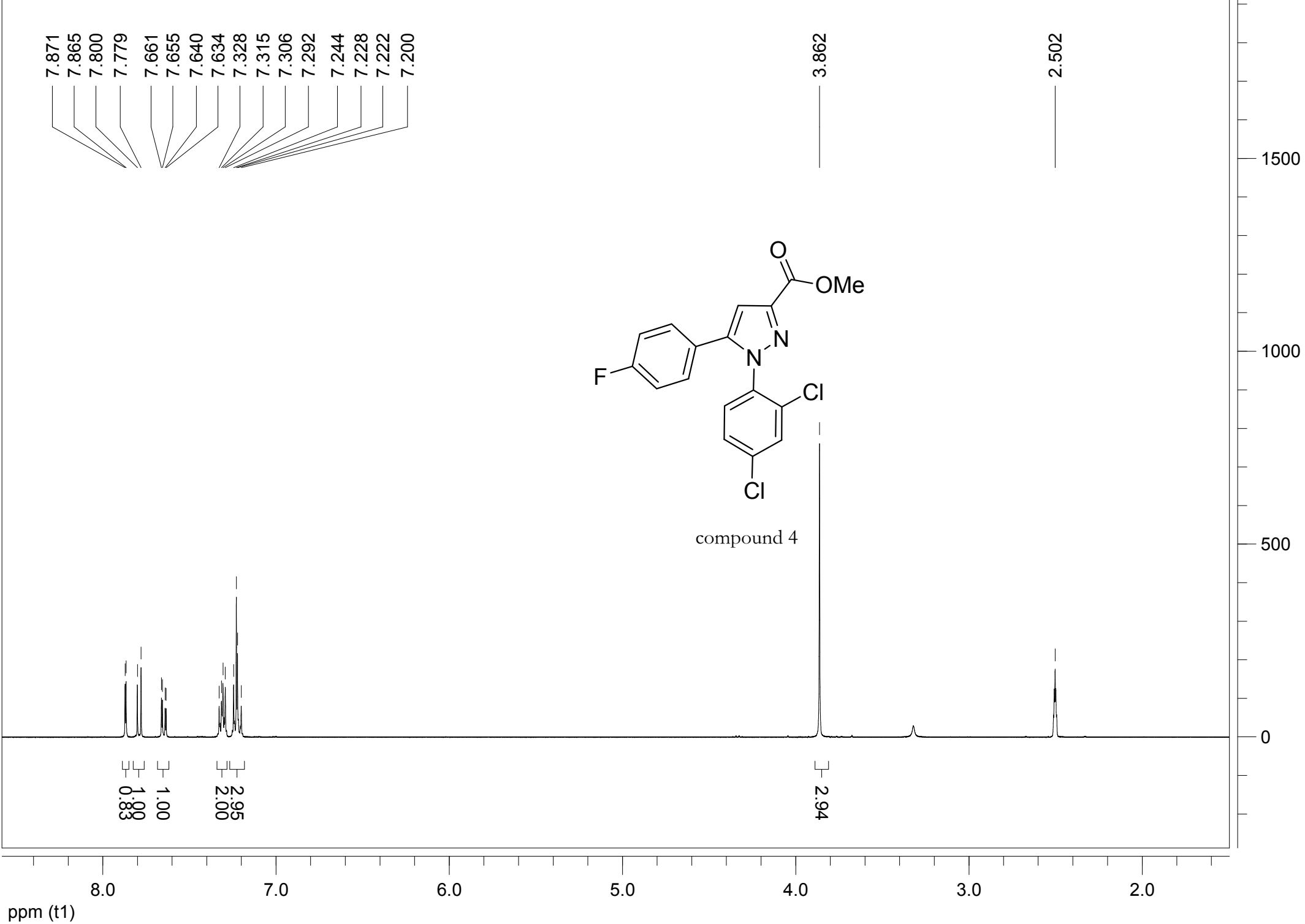

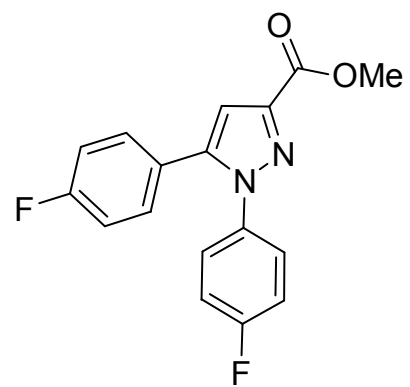

compound 5

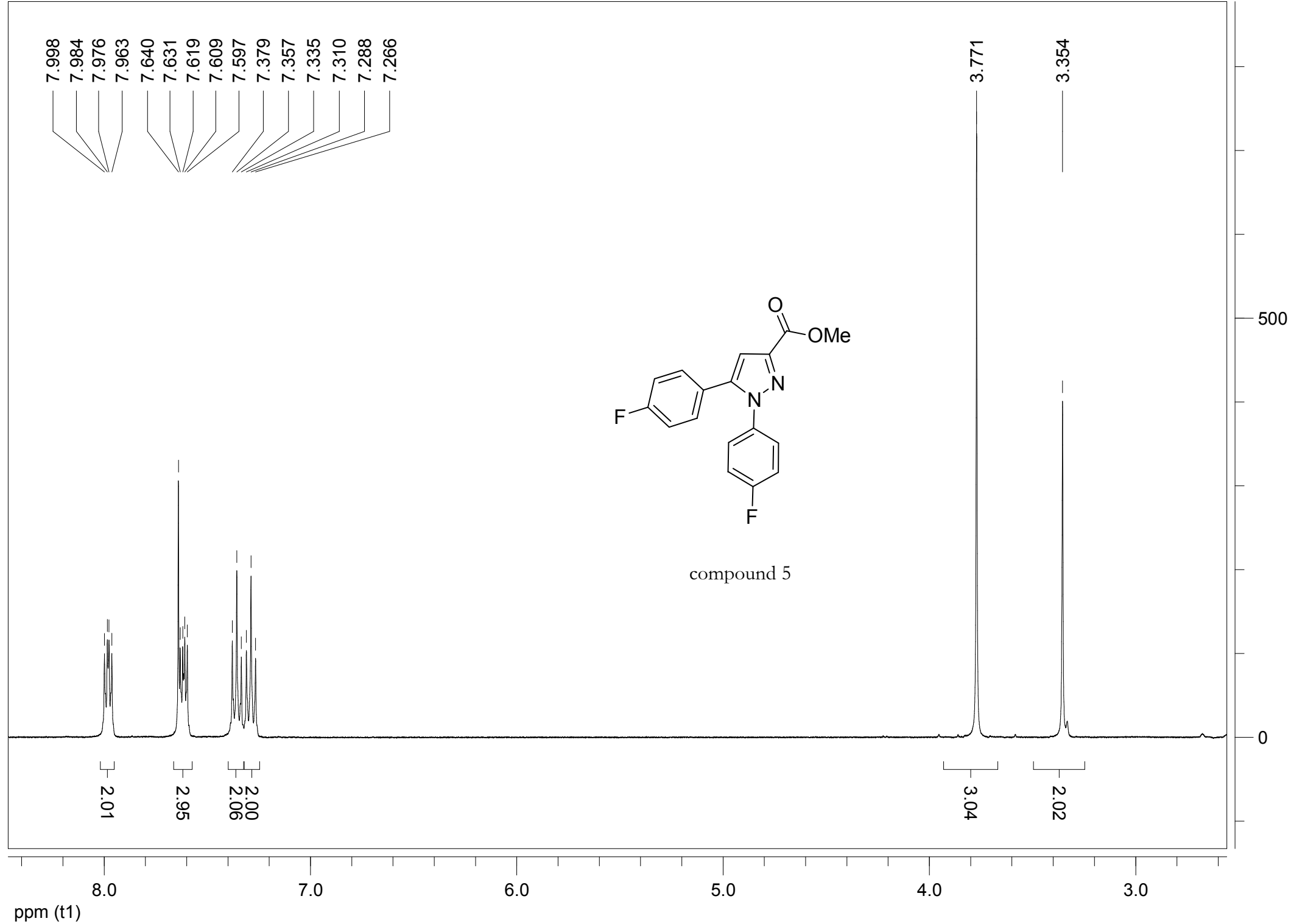

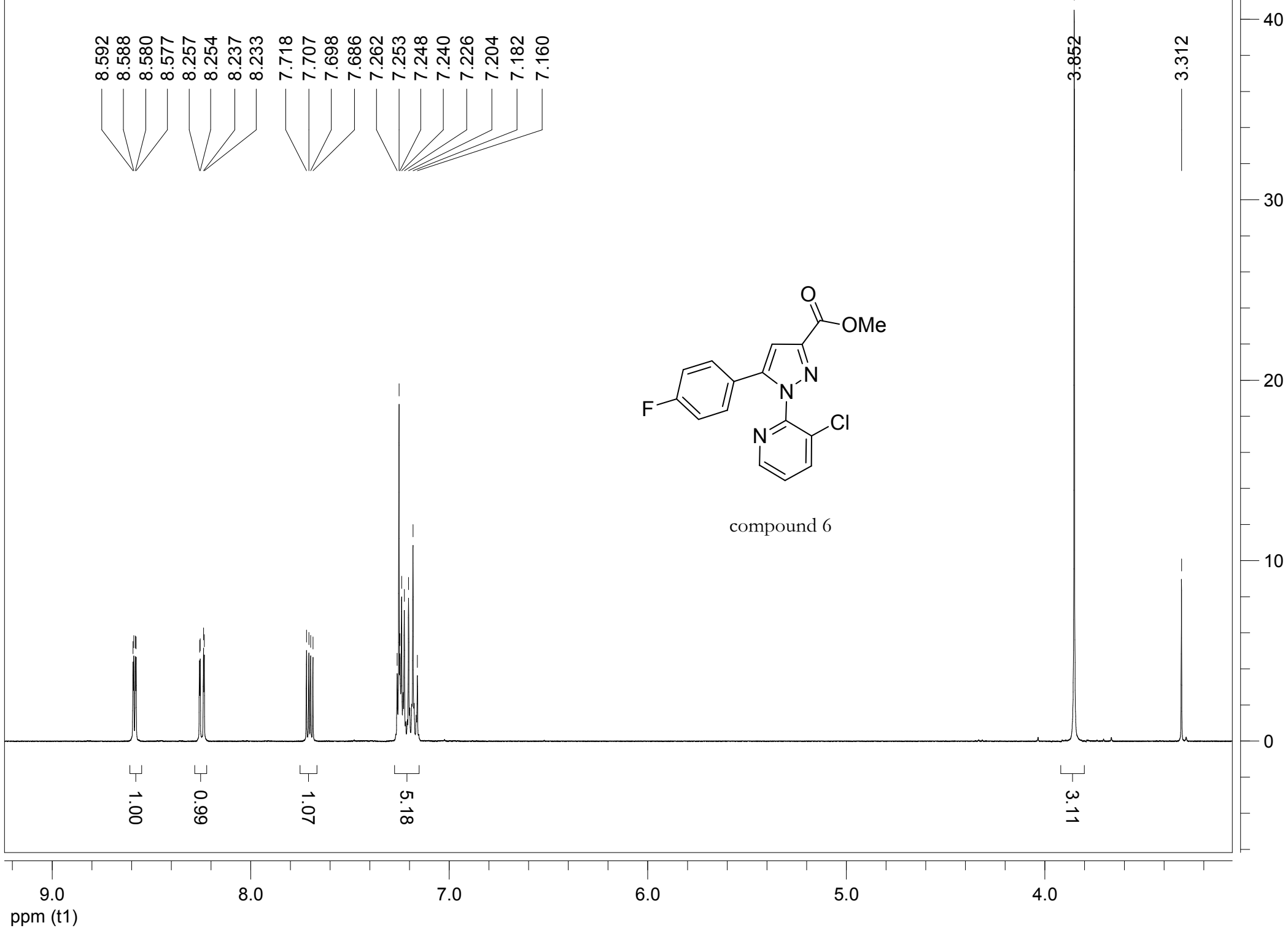

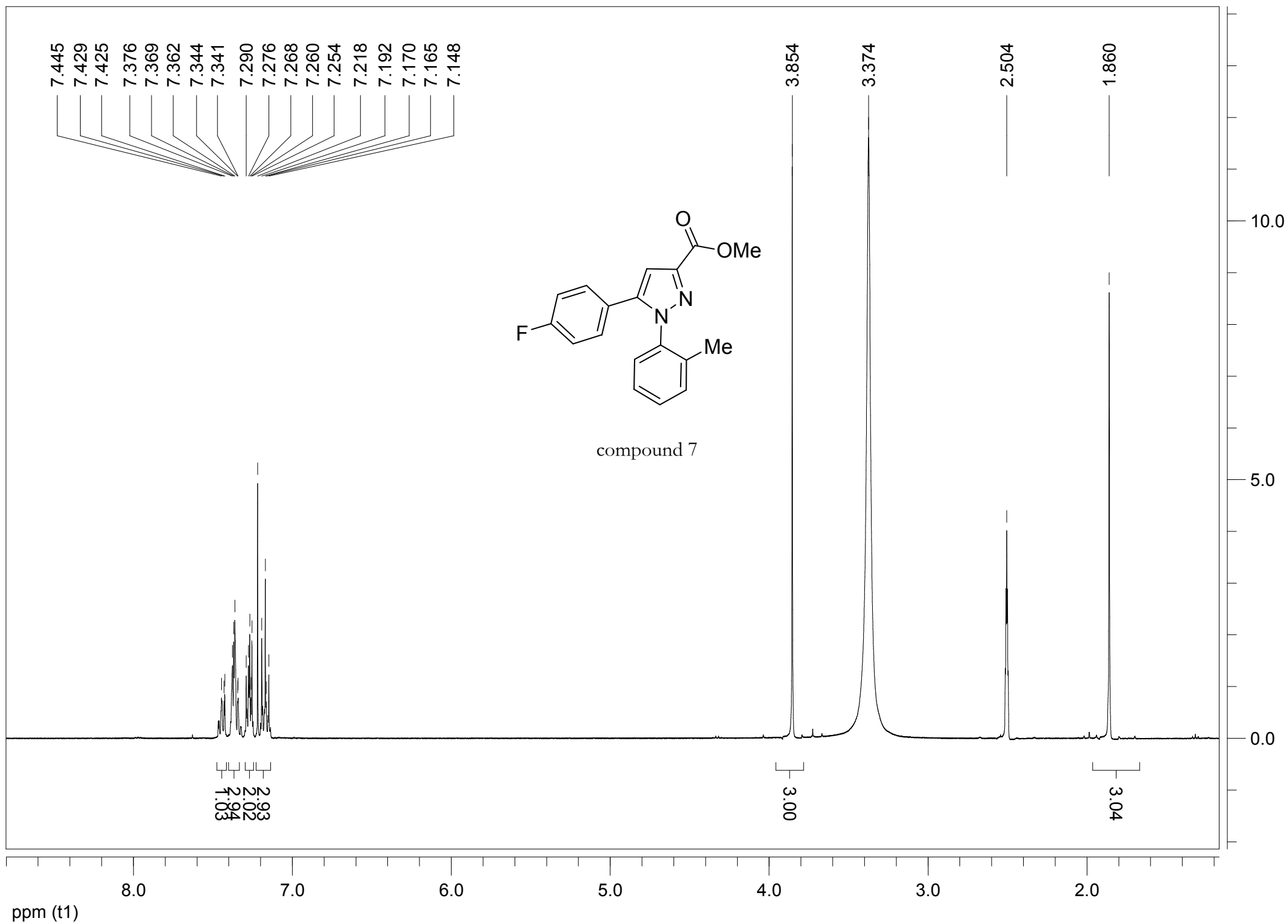

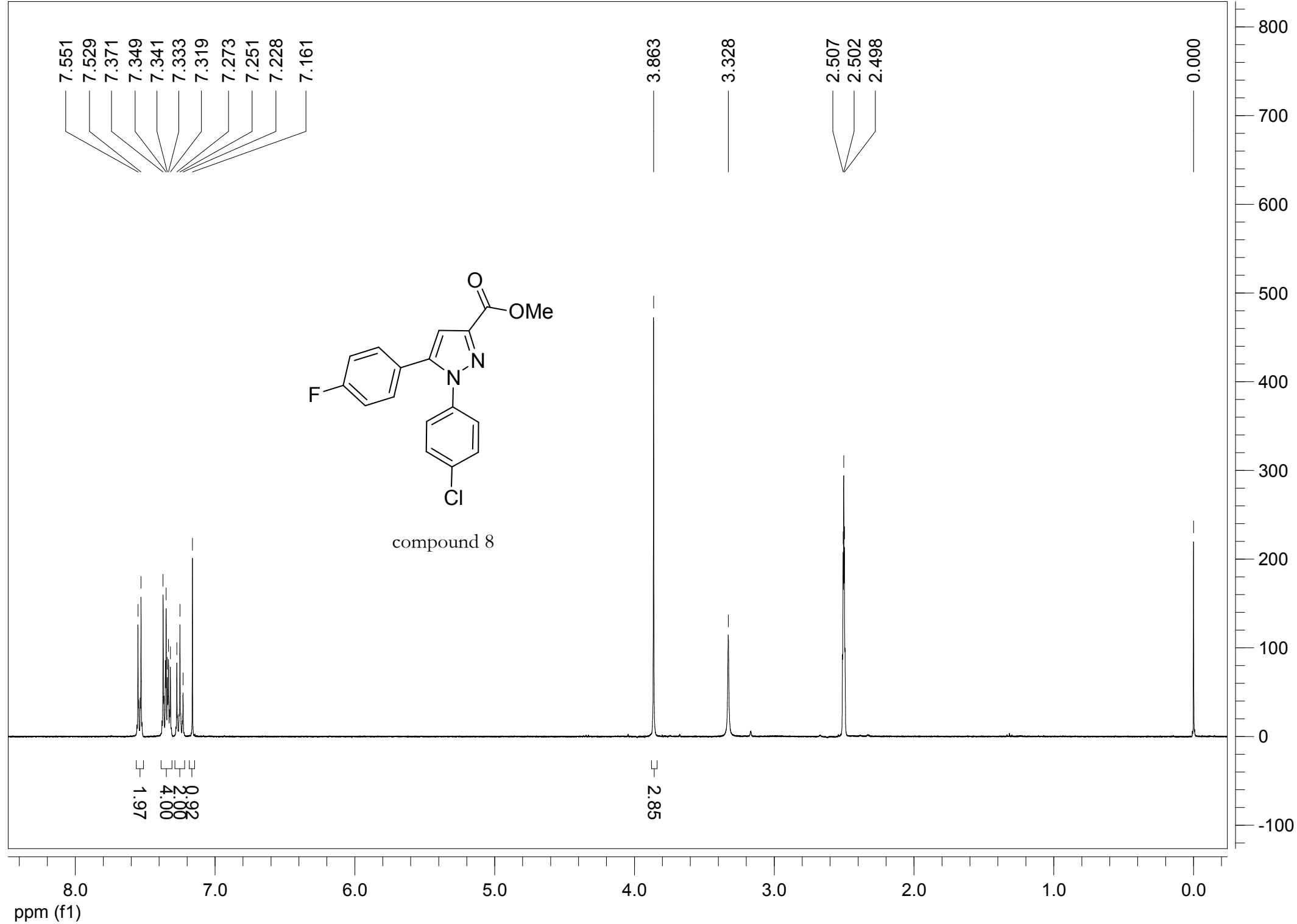

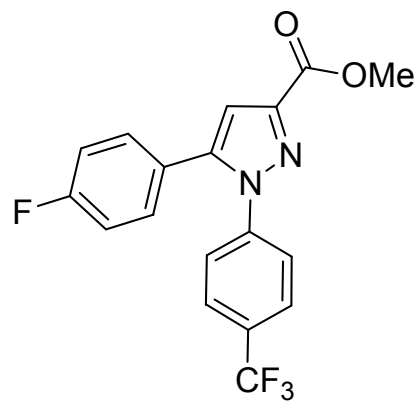

compound 9

7.860  
7.839  
7.569  
7.548  
7.383  
7.369  
7.362  
7.348  
7.287  
7.265  
7.243  
7.189

3.877

3.317

2.507  
2.503  
2.499

0.000

2000

1500

1000

500

0

2.18  
2.16  
2.14  
2.12  
2.10

3.16

5.0

0.0

ppm (t1)

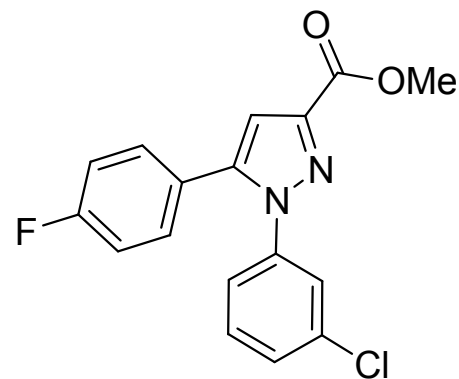

compound 10

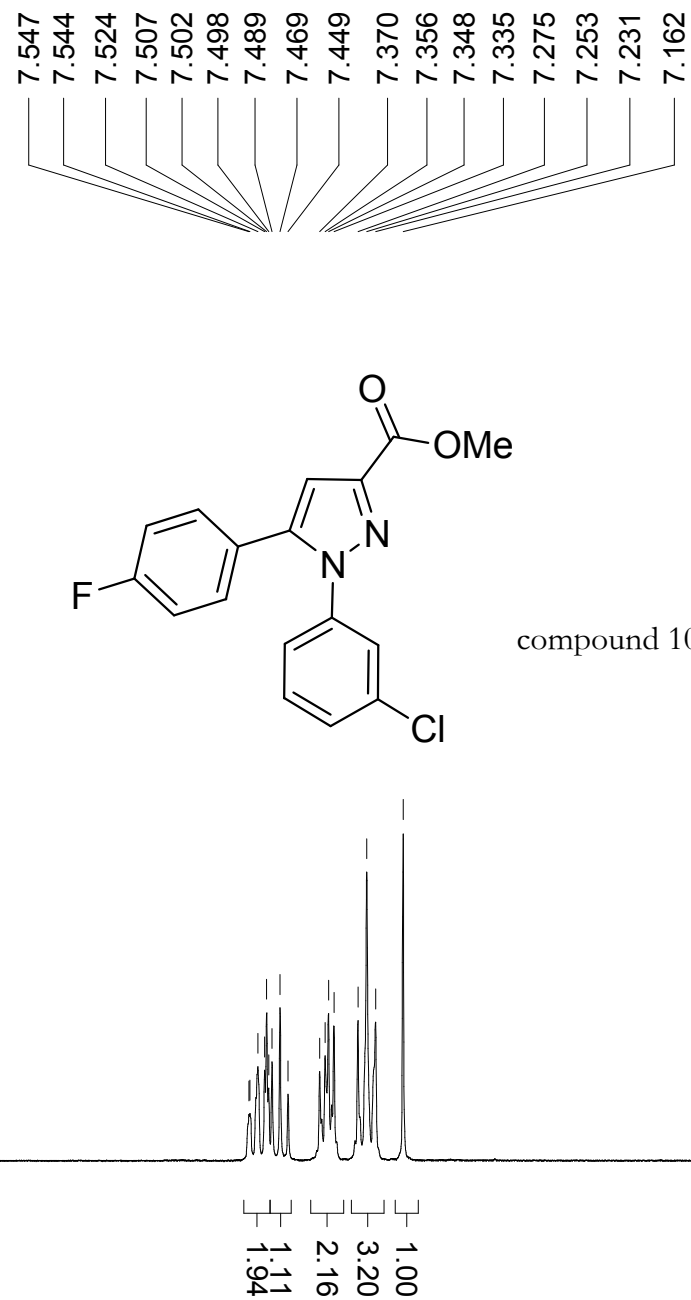

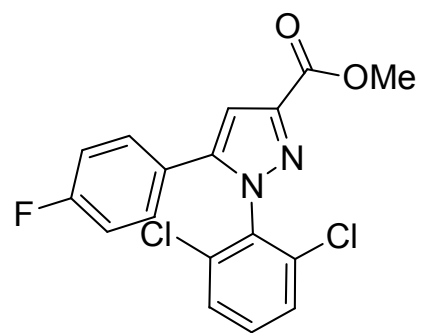

compound 11

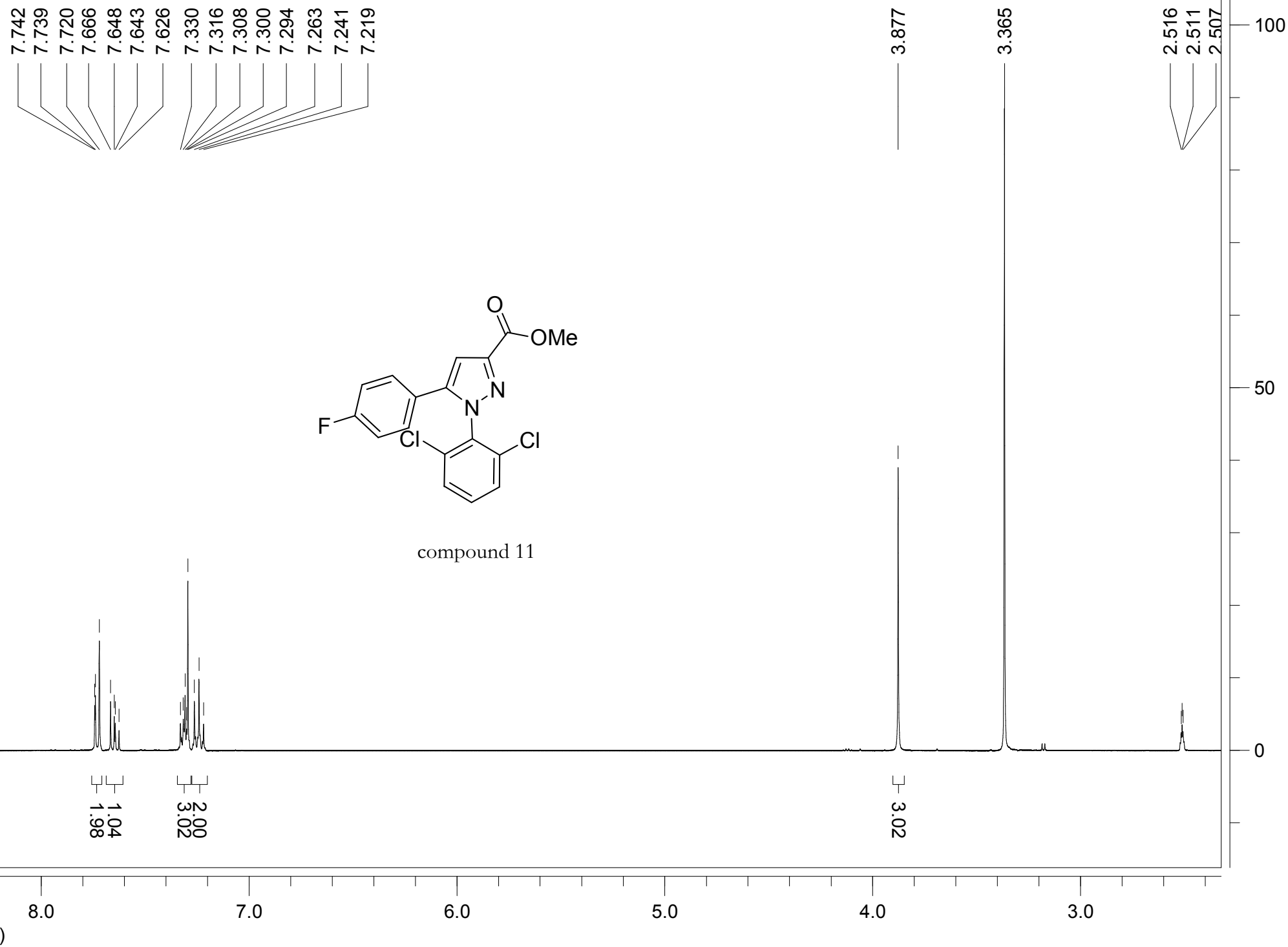

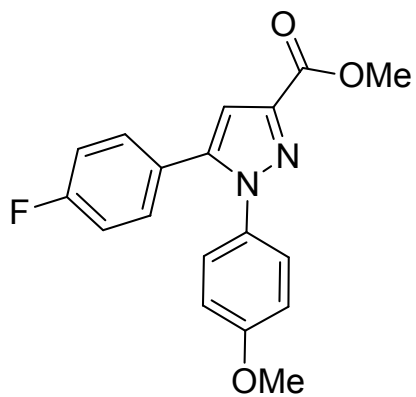

compound 12

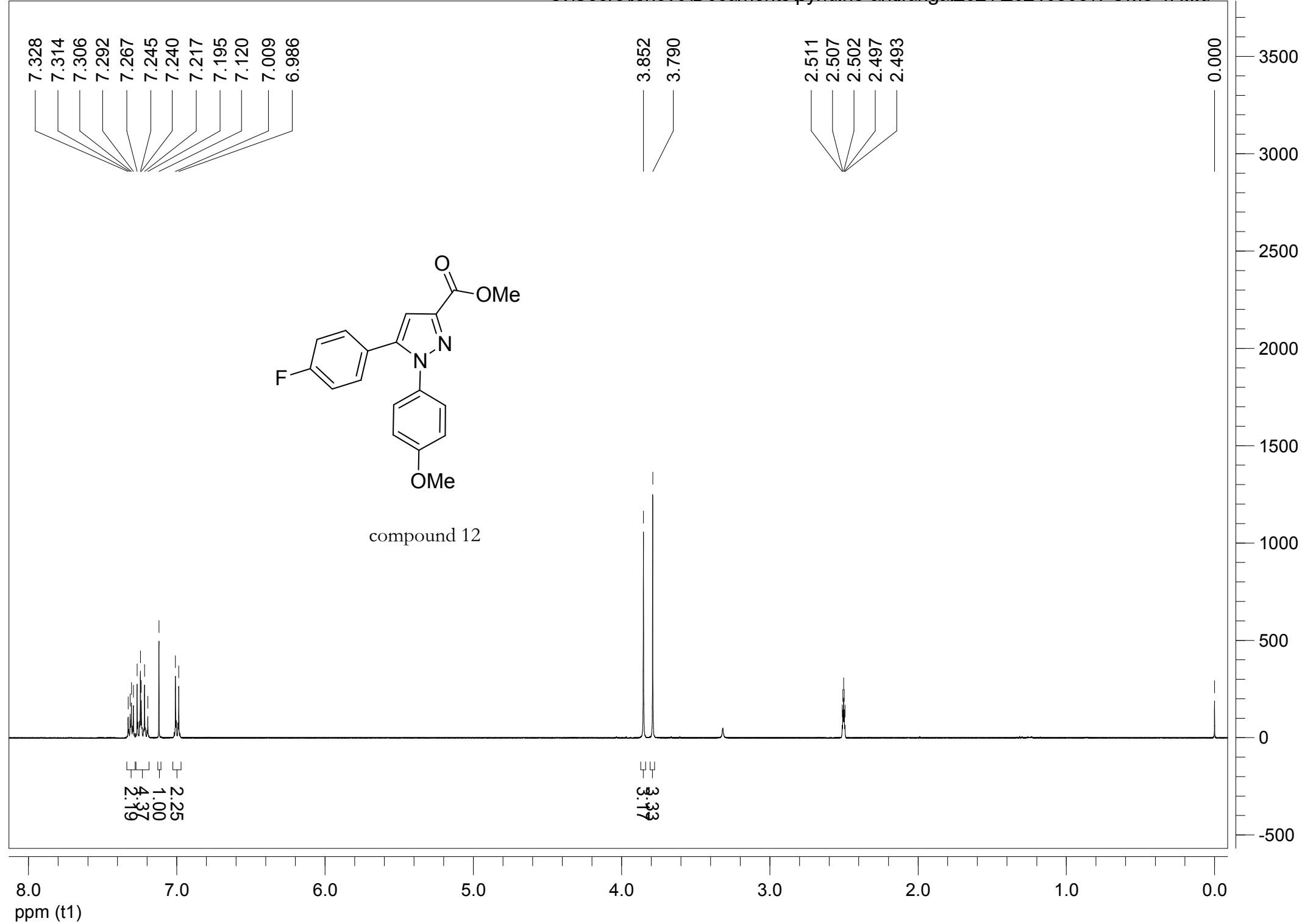

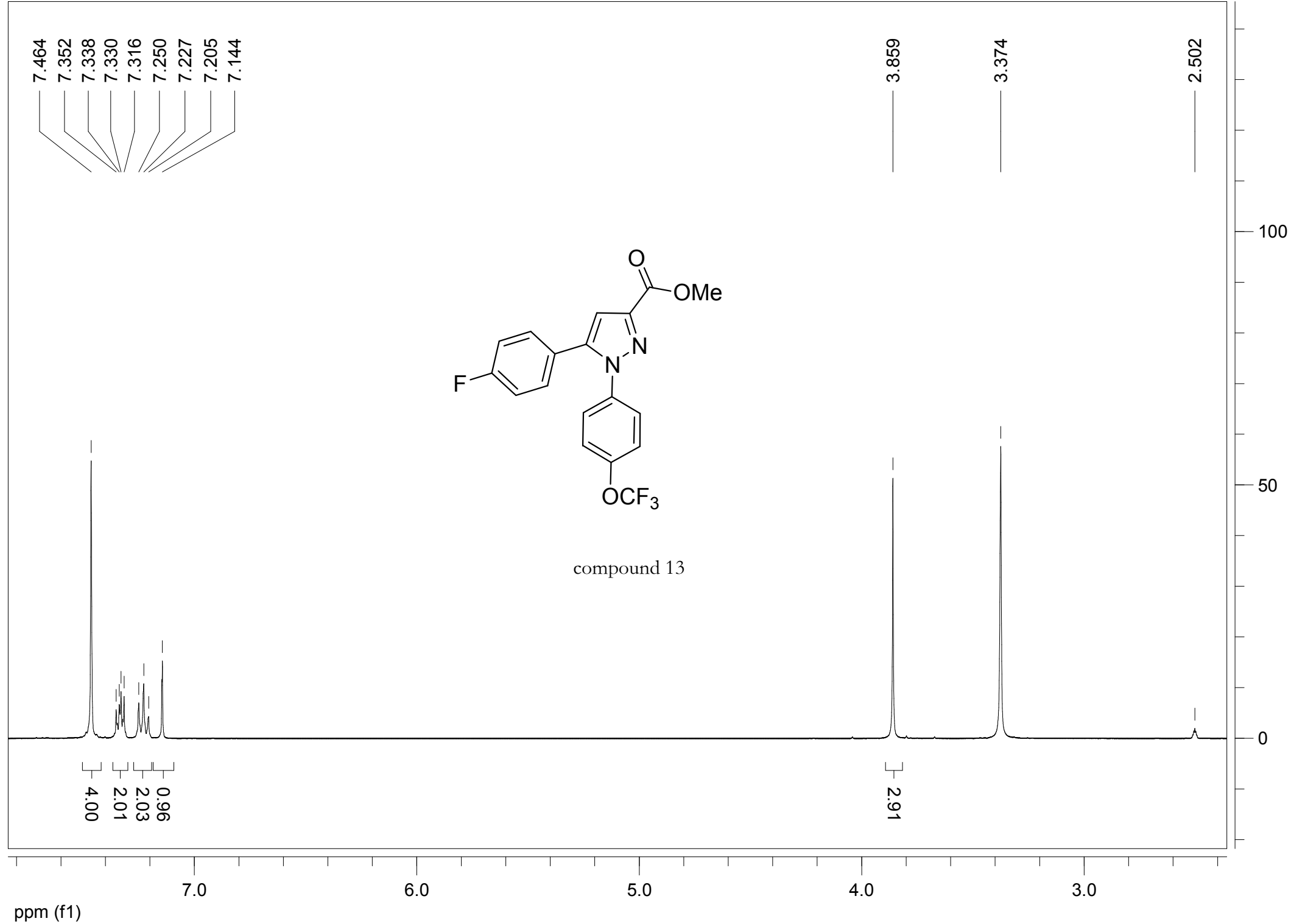

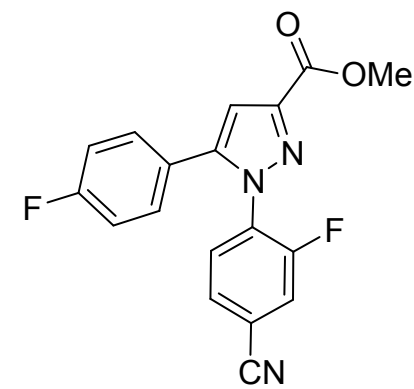

compound 14

8.094  
8.071  
8.067  
7.915  
7.900  
7.342  
7.328  
7.320  
7.306  
7.236  
7.217  
7.195

3.852  
3.333  
2.483

100

50

0

56.0  
66.1

10.2  
2.96

3.00

9.0

8.0

7.0

6.0

5.0

4.0

3.0

2.0

1.0

ppm (t1)

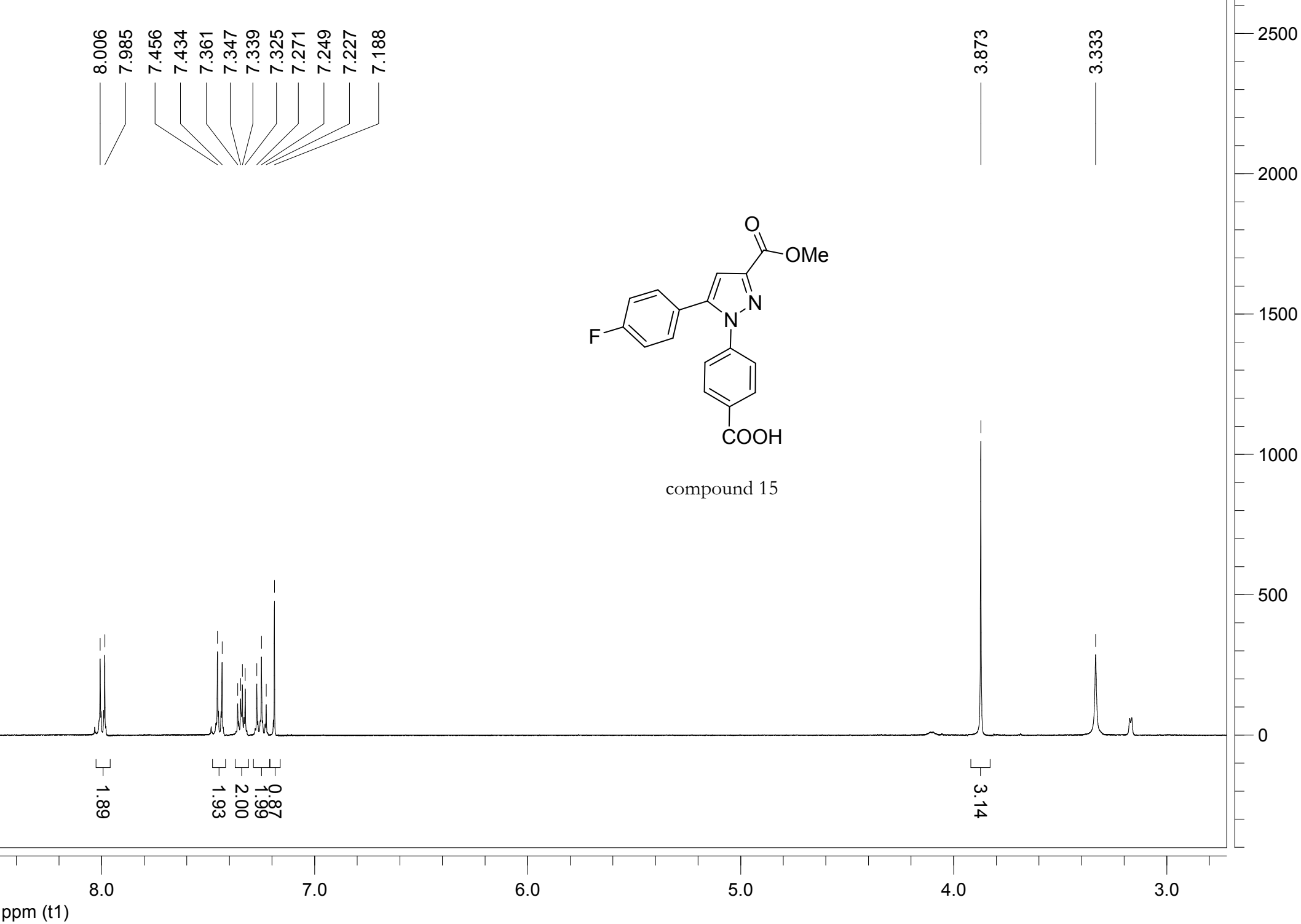

Supplement: Supplementary file 3 [file Data_Sheet_3.PDF]

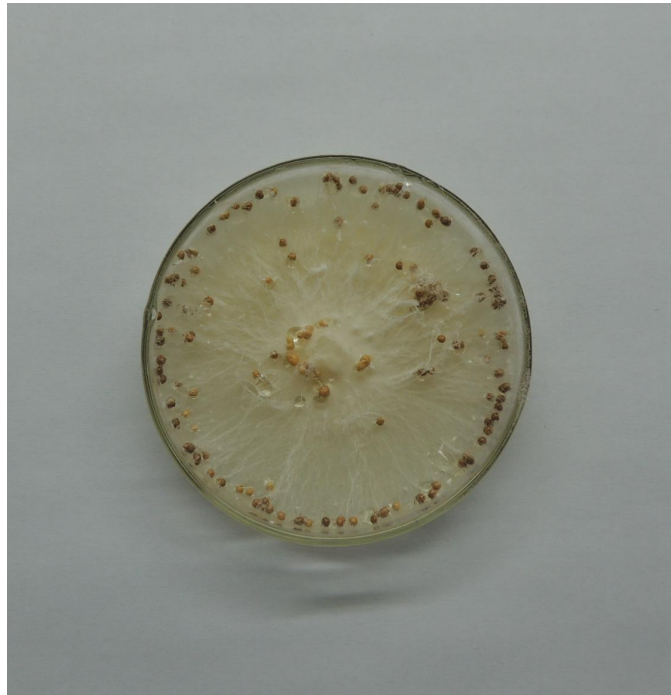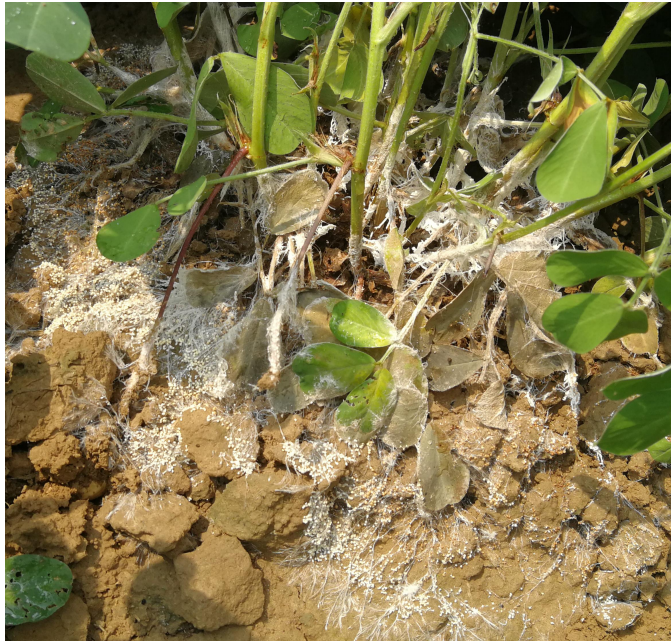

**The morphological characteristics of our isolate in Petri dishes (above) and in the field (below).**

Supplement: Supplementary file 5 [file Data_Sheet_5.PDF]
