# Supplementary material for: Development of: 1,5-Diaryl-Pyrazole-3-Formate Analogs as Antifungal Pesticides and Their Application in Controlling Peanut Stem Rot Disease
Source: Front Microbiol. 2022 Jan 4;12:728173. doi: 10.3389/fmicb.2021.728173 (PMC8763808; doi:10.3389/fmicb.2021.728173)
Supplement: Supplementary file 4 [file Data_Sheet_4.PDF]

Table S1. Inhibition rate of compounds 1-15 against selected pythopathogenic fungi at 100 μg/ml

Compound	% Growth Inhibtion				
	Fusarium oxysporum.f.sp.niveum	*Fusarium graminearium Sehw*	*Phytopthora sp.*	*maydis Nisik & Miy.*	*Myrothecium roridum*
1	5%	16%	5%	14%	20%
2	5%	6%	0%	29%	14%
3	33%	13%	26%	26%	20%
4	31%	19%	28%	31%	27%
5	0%	3%	5%	23%	17%
6	70%	42%	56%	69%	78%
7	53%	32%	47%	49%	3%
8	44%	19%	33%	37%	43%
9	8%	6%	2%	11%	3%
10	8%	3%	2%	14%	7%
11	6%	10%	2%	17%	11%
12	36%	13%	30%	26%	6%
13	11%	0%	12%	6%	26%
14	42%	16%	37%	89%	56%
15	11%	3%	5%	29%	21%
Tebuconazole	100%	100%	100%	100%	97%
Thifluzamide	17%	10%	12%	34%	29%

As shown in table S1, compounds 1-15 along with tebuconazole and thifluzamide were assessed for their antifungal activity against some other fungal pathogens. The compounds exhibited differential inhibitory effects against different phytopathogenic fungi. Generally, compound **6** demonstrated higher activity against all the tested pathogens with inhibition rates of 42%-78% except for the fungus *Helminthosporium maydis Nisik & Miy.*, against which compound **14** showed the highest inhibtion of 89% among all the analogs. Tebuconazole almost completely inhibited the growth of all five tested pathogens as it did with *Sclerotium rolfsii*. In contrast, while thifluzamide is even more active against *Sclerotium rolfsii* than tebuconazole, it did not exhibit obvious antifungal effects against the the five fungi listed in the table S1.
